# Hepatic Hedgehog signaling contributes to the regulation of IGF1 and IGFBP1 serum levels

**DOI:** 10.1186/1478-811X-12-11

**Published:** 2014-02-18

**Authors:** Madlen Matz-Soja, Susanne Aleithe, Eugenia Marbach, Jan Böttger, Katrin Arnold, Wolfgang Schmidt-Heck, Jürgen Kratzsch, Rolf Gebhardt

**Affiliations:** 1 Institute of Biochemistry, Faculty of Medicine, University of Leipzig, Leipzig, Germany; 2 Leibniz Institute for Natural Product Research and Infection Biology – Hans Knoell Institute, Jena, Germany; 3 Institute of Laboratory Medicine, Clinical Chemistry and Molecular Diagnostics, University of Leipzig, Leipzig, Germany

**Keywords:** *Gli3*, Growth hormone, Hedgehog signaling, Hepatocytes, IGF-I, IGFBP-1, Liver, MotifMap, *Patched1*, *Smoothened*

## Abstract

**Background:**

Hedgehog signaling plays an important role in embryonic development, organogenesis and cancer. In the adult liver, Hedgehog signaling in non-parenchymal cells has been found to play a role in certain disease states such as fibrosis and cirrhosis. However, whether the Hedgehog pathway is active in mature healthy hepatocytes and is of significance to liver function are controversial.

**Findings:**

Two types of mice with distinct conditional hepatic deletion of the *Smoothened* gene, an essential co-receptor protein of the Hedgehog pathway, were generated for investigating the role of Hedgehog signaling in mature hepatocytes. The knockout animals (KO) were inconspicuous and healthy with no changes in serum transaminases, but showed a slower weight gain. The liver was smaller, but presented a normal architecture and cellular composition. By quantitative RT-PCR the downregulation of the expression of Indian hedgehog (*Ihh*) and the *Gli3* transcription factor could be demonstrated in healthy mature hepatocytes from these mice, whereas *Patched1* was upregulated. Strong alterations in gene expression were also observed for the IGF axis. While expression of *Igf1* was downregulated, that of *Igfbp1* was upregulated in the livers of both genders. Corresponding changes in the serum levels of both proteins could be detected by ELISA. By activating and inhibiting the transcriptional output of Hedgehog signaling in cultured hepatocytes through siRNAs against *Ptch1* and *Gli3*, respectively, in combination with a ChIP assay evidence was collected indicating that *Igf1* expression is directly dependent on the activator function of *Gli3*. In contrast, the mRNA level of *Igfbp1* appears to be controlled through the repressor function of *Gli3*, while that of *Igfbp2* and *Igfbp3* did not change. Interestingly, body weight of the transgenic mice correlated well with IGF-I levels in both genders and also with IGFBP-1 levels in females, whereas it did not correlate with serum growth hormone levels.

**Conclusions:**

Our results demonstrate for the first time that Hedgehog signaling is active in healthy mature mouse hepatocytes and that it has considerable importance for IGF-I homeostasis in the circulation. These findings may have various implications for mouse physiology including the regulation of body weight and size, glucose homeostasis and reproductive capacity.

## Lay abstract

### Background

The Hedgehog signaling pathway, like that of other morphogens, plays an important role in embryonic development, organogenesis and tissue homeostasis. In the adult stage, its activity is downregulated in most organs, but can be reactivated in physiological and pathological processes such as tissue regeneration and cancer. In the adult liver, Hedgehog signaling is lower in parenchymal cells, the hepatocytes, than in non-parenchymal cells where it has been found to play a fundamental role in certain disease states such as fibrosis and cirrhosis. Whether the Hedgehog pathway is active in mature healthy hepatocytes and is of significance to liver function are controversial.

### Findings

In order to approach these questions we used two types of mice with distinct conditional hepatic deletion of the *Smoothened* gene, an essential co-receptor protein of the Hedgehog pathway. Knockout of this gene interrupted the Hedgehog pathway only in hepatocytes. The knockout mice were healthy, but showed a slower gain of body and liver weight. Alterations in the expression of Hedgehog signaling components revealed the functional activity of this pathway in the liver. Strong alterations in gene expression were also observed for insulin-like growth factor-I (IGF-I) the serum level of which correlated well with body weight.

### Conclusions

Our results demonstrate for the first time that Hedgehog signaling is active in mature mouse hepatocytes and that it has considerable importance for IGF-I homeostasis in the circulation. These findings may have various implications for mouse physiology including the regulation of body weight and size, glucose homeostasis and reproductive capacity.

## Background

Hedgehog (Hh) signaling is known to play an important role in embryonic development, organogenesis and cancer [1,2]. Secreted Hh ligands (Sonic hedgehog (SHH), Indian hedgehog (IHH) and Desert hedgehog (DHH)) influence on target cells via autocrine, paracrine or even endocrine-like ways. They bind to PTCH1 and PTCH2 receptors and alleviate Patched-mediated suppression of Smoothened (SMO), a putative seven-transmembrane protein with features of a G-protein coupled receptor [3,4]. Activated *Smo* induces a number of downstream reactions, finally leading to stabilization of Gli transcriptional factors, their translocation into nucleus and transcription of Hh-dependent genes. In vertebrates three Gli genes are present. GLI1 is acting predominantly as a transcriptional activator of Hh target genes, while GLI2 and GLI3 may act as both activators and repressors depending on Hh activity [5,6].

In adult tissues Hh signaling is commonly downregulated and may be reactivated only in certain types of cancer [7,8]. Like for other morphogens, however, Hh signaling in adult tissues frequently shows activity gradients that are involved in maintenance of tissue homeostasis. Well studied examples are the maintenance of the specific phenotype of gastric and intestinal epithelium [9,10] and the fate of adipose tissue [11,12]. In the adult liver, active Hh signaling has been observed in some non-parenchymal cells namely hepatic stellate cells (HSC) and cholangiocytes [13,14], where it plays a fundamental role in maintaining the normal phenotype of these cells but also during their activation in certain disease states such as fibrosis and cirrhosis [14]. Under diseased conditions also stressed or dying hepatocytes (e.g. when ballooning in severe liver steatosis) were described to secret high amounts of Shh which appears to act as a major signal for inducing alternative regenerative pathways via stem cell populations [15-17]. However, whether the Hh signaling pathway is active in healthy, mature hepatocytes and relevant to normal liver function remains controversial [13,18]. On the one hand, a significant contribution of Hh signaling to the regulation of liver function has been denied because of the low expression level of the respective signaling components in mature hepatocytes compared with certain types of liver cancer cells or even non-parenchymal cells such as HSC or cholangiocytes [13,14]. On the other hand, theoretical considerations based on analogy with other tissues considerably support the hypothesis that Hh signaling might indeed play a fundamental role in controlling liver function [18].

In order to clarify the important question of whether Hh signaling is active in healthy, mature hepatocytes, we have chosen two independent types of transgenic mice with conditional hepatocellular ablation of *Smoothened*, a bottleneck component of the Hh pathway [19], to interrupt Hh signaling in hepatocytes. These mice should be optimally suited for investigating the functional relevance, if any, of low Hh signaling in these cells. To exclude any aberrant findings due to damaged or immature hepatocytes we carefully checked the histological integrity of the livers after knockout of *Smo* including the integrity and phenotype of the hepatocytes *in situ*. A second method for gaining insight into the functional relevance of Hh signaling in hepatocytes was to use RNAi experiments *in vitro* for modulating (i. e. inhibiting or activating) Hh signaling and monitoring the altered transcriptional and functional output. For that purpose a high purity of the cultured hepatocytes was guaranteed. Using these approaches and various analytical techniques we were able to demonstrate for the first time to our knowledge that Hh signaling is active in mature hepatocytes and contributes to the regulation of IGF-I and IGFBP-1 protein serum levels in mice.

## Results

### Generation and phenotypic characteristics of Smo-KO mice

For investigating the influence of the Hedgehog signaling pathway on liver function, hepatocyte-specific ablation of *Smo*, a bottleneck component of Hh signaling [19], seemed a suitable approach. In the first type of transgenic mice, conditional ablation of *Smo* in liver epithelial cells was induced by mating *Smo*^flox/flox^ and Alfp-Cre mouse lines (Additional file 1: Figure S1A-C). The resulting double transgenic homozygous *Smo*^-/-^ mice are abbreviated as SAC-KO mice. Alfp-Cre mice [20] have been successfully used for liver-specific knockout studies [21]. In agreement with these studies, Cre-recombinase could be detected by immunohistochemistry in all liver epithelial cells (hepatocytes and cholangiocytes) (Additional file 1: Figure S1D), and only the recombined *Smo* alleles could be detected in the livers of homozygous mice after 12 weeks (Additional file 1: Figure S1E). As expected, significant loss of *Smo* expression was found in the liver and in isolated hepatocytes, and no changes were found in the heart, lung, spleen, adipose tissue or kidney (Additional file 1: Figure S1F).

**Figure 1:**
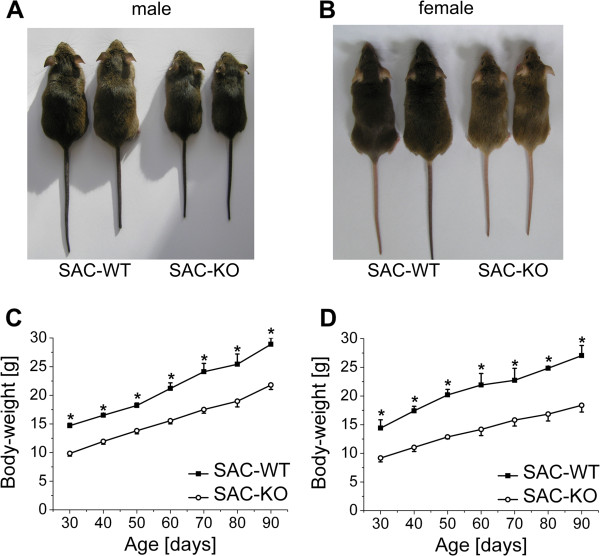
**Phenotypic features induced by hepatocyte-specific ablation of Smoothened in transgenic SAC mice.** Photographs of **(A)**: male SAC mice and **(B)**: female SAC mice show two WT mice (left) and two KO mice (right). Comparison of body weight of **(C)**: male SAC-WT (black squares) (n = 4) and SAC-KO (open circles) (n = 5) mice and **(D)**: female SAC-WT (black squares) (n = 3) and SAC-KO (open circles) (n = 4) mice. Values are presented as the means ± SEM; *, p < 0.05.

The first visible phenotype of the SAC-KO mice was evident immediately after birth; the mice were small in size compared to their WT littermates (Figure 1A, B). At 30 days after birth, when the offspring were separated from their dams, body weight was significantly reduced in both male and female homozygous SAC-KO mice compared to gender-matched WT mice (Figure 1C, D). Weight gain in the transgenic males paralleled that in WT males up to the age of 12 weeks, after which it appeared to progress at a slightly lower pace in transgenic female mice during the same period of time (Figure 1D). Interestingly, the weight of the heterozygous SAC-KO mice (*Smo*^+/-^) was significantly different from that of both, the WT littermates and the homozygous mice (Additional file 2: Figure S2A,B) indicating that the loss of each *Smo* allel caused a distinct drop of body weight. The age of 12 weeks was chosen for further investigations because the animals were fully mature at this time point, which allowed the proper isolation of the mature hepatocytes essential for this study.

Generally, the SAC-KO mice were healthy and their behavior was inconspicuous. They showed slightly reduced blood glucose levels in the postprandial state and had significantly lower plasma insulin levels (Table 1).

**Table 1:** Liver parameters and serum activities of male and female SAC-WT and SAC-KO mice

**Male mice**	**SAC-WT**	**n**	**SAC-KO**	**n**	**p value (MWU-test)**
Liver weight (g)	1.65 ± 0.09	6	0.95 ± 0.03	14	0.001**
Liver-body-weight ratio (%)	5.58 ± 0.16	6	4.38 ± 0.14	14	0.001**
ALAT (μkat/l)	1.64 ± 0.48	3	2.47 ± 0.37	4	0.228
ASAT (μkat/l)	8.07 ± 3.97	3	6.82 ± 1.83	4	1.000
GLDH (μkat/l)	0.35 ± 0.08	3	1.93 ± 0.58	4	0.057
Insulin (pmol/l)	160.53 ± 43.84	7	44.86 ± 11.97	5	0.0101*
Glucose (mMol/dl)	8.12 ± 0.48	5	7.22 ± 0.40	7	0.34
**Female mice**					
Liver weight (g)	1.29 ± 0.06	17	1.10 ± 0.06	8	0.724
Liver-body-weight ratio (%)	5.82 ± 0.18	17	4.91 ± 0.16	8	0.001**
ALAT (μkat/l)	1.48 ± 0.50	6	2.47 ± 0.37	6	0.228
ASAT (μkat/l)	4.80 ± 0.44	6	7.07 ± 1.10	6	1.000
GLDH (μkat/l)	0.54 ± 0.10	6	1.04 ± 0.28	6	0.057
Insulin (pmol/l)	98.24 ± 10.68	10	56.73 ± 5.89	8	0.0021**
Glucose (mMol/dl)	7.19 ± 0.24	9	6.22 ± 0.40	10	0.079

Determination of body weight and liver parameters at the age of 12 weeks. Values represent means ± SEM; *, p < 0.05; **, p < 0.01.

**Figure 2:**
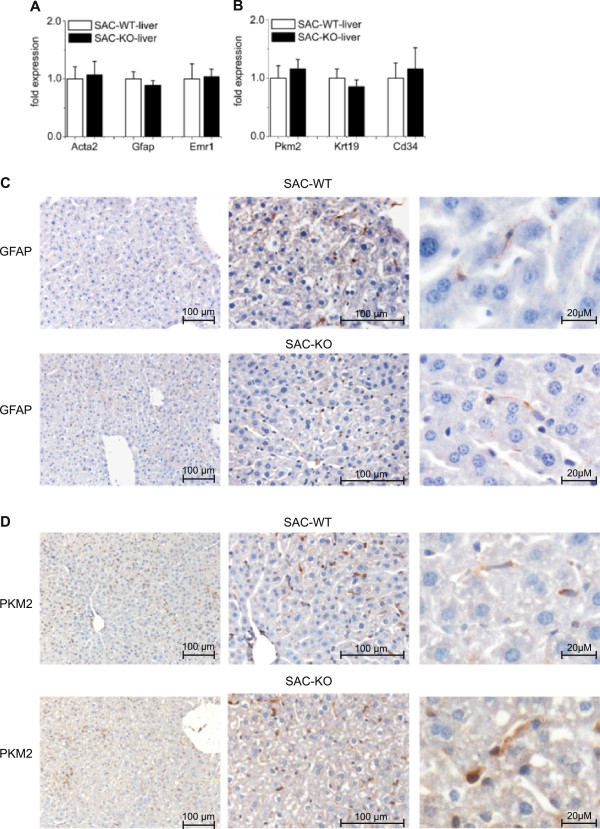
**Expression of markers for non-parenchymal cells in transgenic SAC mice.** qRT-PCR analyses of **(A)**: *Acta2*, *Gfap* and *Emr1*; **(B)**: *Pkm2*, *Krt19* and *Cd34* in liver tissue of 12 weeks old male SAC-WT (white bars) (n = 13) and SAC-KO (black bars) (n = 11) mice. Values are presented as the means ± SEM. Immunohistochemistry in liver sections from male SAC-WT and SAC-KO mice of **(C)**: GFAP (present only in HSC’s), **(D)**: PKM2 (Is present in all non-parenchymal cell types. Hepatocytes in SAC-KO mice show a very light cytoplasmic staining). Bar: 100 μm and 20 μM.

Histological examination of the liver revealed a normal lobular architecture, indicating that *Smo* ablation did not influence the correct development of this organ despite leading to a smaller size. Liver weight was significantly reduced by approx. 40% in male SAC-KO mice (Table 1), and the liver weight/body weight ratio dropped from 5.6% in WT mice to 4.4% in SAC-KO mice. In female mice, similar alterations were observed, except for a smaller difference in total liver weight (Table 1). Apart from the slower liver weight/body weight ratio compared to SAC-WT mice, there were no indications of overt liver damage in the SAC-KO mice in both genders. Accordingly, no significant differences could be observed in serum activities of ASAT, ALAT and GLDH between SAC-WT and SAC-KO mice (Table 1). Furthermore, comparing the expression levels of different marker genes for hepatic stellate cells (HSC) and myofibroblasts (*Gfap, Acta2*) or Kupffer cells (*Emr1*), in liver tissue from SAC-WT mice and SAC-KO mice revealed no major changes in the cellular composition (Figure 2A, B). This could be confirmed by immunohistochemistry as exemplified by the distribution of GFAP which was found only in HSC and cholangiocytes (Figure 2C). Only the general marker of non-parenchymal cells, PKM2 [22] seemed to be slightly induced in hepatocytes from SAC-KO mice (Figure 2D).

### Influence of *Smoothened* ablation on Hedgehog pathway components in hepatocytes *in situ* and *ex vivo*

**Figure 3:**
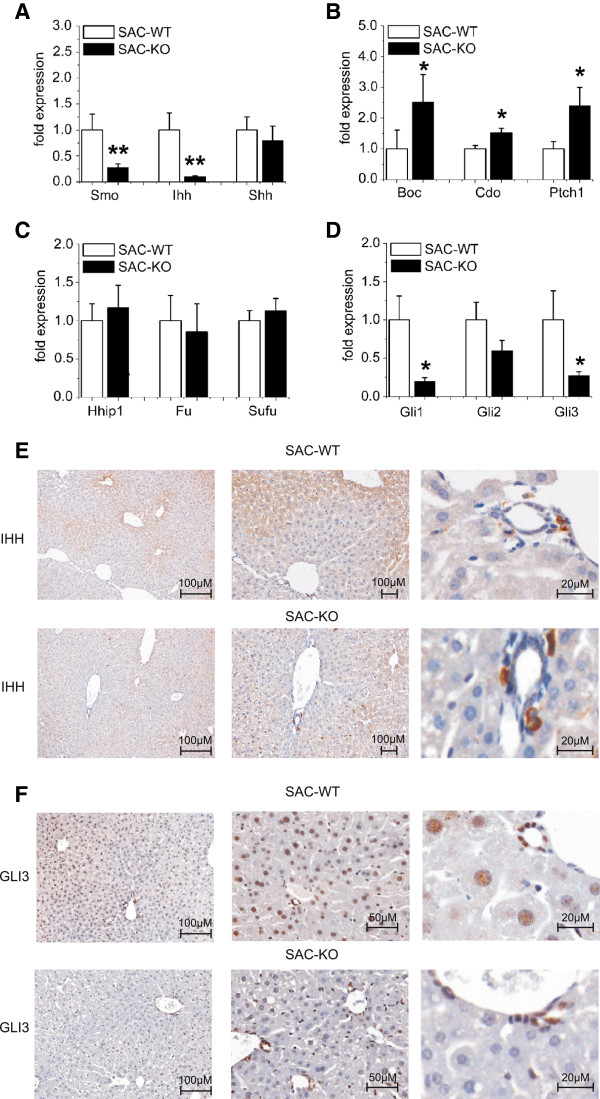
**Expression changes of Hedgehog signaling components.** qRT-PCR analyses of hedgehog members in hepatocytes freshly isolated from 12-week-old male SAC-WT (white bars) (n = 8-17) and SAC-KO (black bars) (n = 8-13) mice: **(A)**: *Smo*, *Ihh* and *Shh*; **(B)**: *Boc*, *Cdo* and *Ptch1*; **(C)**: *Hhip1*, *Fu* and *Sufu*, and the transcription factors **(D)**: *Gli1*, *Gli2* and *Gli3.* Values are presented as relative means ± SEM; *, p < 0.05, **, p < 0.01. Immunohistochemical staining of **(E)**: Indian Hedgehog and **(F)**: GLI3 in liver sections from male transgenic SAC-WT and SAC-KO mice at the age of 12 weeks. Scale bars: 100 μM; 50 μM and 25 μM.

To analyze hepatocellular Hh signaling, the expression of several components involved in the pathway was determined by qRT-PCR in freshly isolated hepatocytes (Figure 3). Purity of the hepatocyte preparation, prepared as described in Materials and Methods, was carefully checked using the markers for hepatocytes (*Arg1, Gck, Gls2, Ldha, Krt8, Krt18*) (Additional file 3: Figure S3A,B). For non-parenchymal cells like hepatic stellate cells, myofibroblasts and Kupffer cells we used the markers shown above. Furthermore we used markers for cholangiocytes (*Krt19*) and endothelial cells (*Cd34*) (Additional file 3: Figure S3C,D). In particular, contamination of hepatocyte preparations by cholangiocytes was excluded as described previously [23].

Apart from the expected loss of *Smo* expression in hepatocytes, the mRNA level of *Ihh* was significantly downregulated, whereas *Boc*, *Cdo* and *Ptch1* were significantly upregulated (Figure 3A, B). The expression levels of *Shh*, the inhibitor *Hhip1* and components acting further downstream, such as *Fused* and *Sufu*, were not significantly affected (Figure 3C). Of the three members of the Gli family of zinc-finger transcription factors, *Gli1* and *Gli3* were strongly downregulated (p < 0.05, n = 7), whereas *Gli2* appeared to be less affected (Figure 3D). These results were comparable in female mice (not shown) and clearly indicate that Hh signaling is active in mature hepatocytes. These findings are in close agreement with those in other cells challenged by *Smo* knockout [24]. Notably, *Ihh* expression appears to be characteristic of healthy hepatocytes, whereas *Shh* is expressed by damaged hepatocytes [16].

By immunohistochemistry it was possible to demonstrate that indeed hepatocytes *in situ* stained positive for IHH and GLI3 protein in the cytoplasm and the nuclei, respectively (Figure 3E, F). Interestingly, staining for IHH showed a pronounced pericentral distribution. In livers of SAC-KO mice both proteins were downregulated resulting in a weaker cytoplasmic staining (IHH) or a loss of nuclear staining (GLI3) (Figure 3F).

### Ablation of *Smo* in hepatocytes alters serum levels of proteins of the IGF axis

Hepatocytes are the major source of circulating IGF-I in many species including mice [25]. Because we have recently hypothesized that IGF-I might be a possible target of Hh signaling in these cells [18], we set out to evaluate the expression of IGF-I and other members of the IGF axis in hepatocytes freshly isolated from WT and SAC-KO mice.

**Figure 4:**
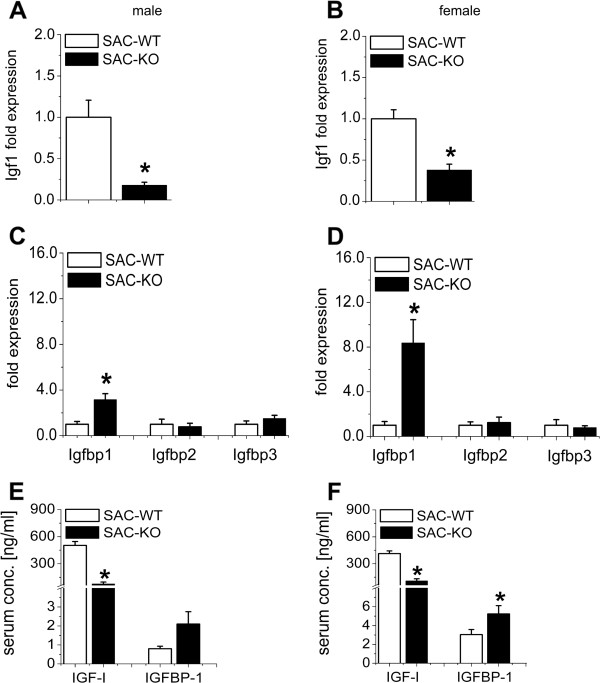
**Changes of IGF axis members in the liver and serum.** Quantitative RT-PCR analyses of **(A, B)**: *Igf1* and **(C, D)**: *Igfbp1*, *Igfbp2* and *Igfbp3* mRNA levels in hepatocytes isolated from 12-week-old male SAC-WT (white bars) (n = 7) and SAC-KO (black bars) (n = 7) (left diagrams) and female SAC-WT (white bars) (n = 7) and SAC-KO (black bars) (n = 5) (right diagrams) mice relative to that of *β-actin.* Values are presented as the means ± SEM; *, p < 0.05. **(E, F)**: Serum analyses of circulating IGF-I and IGFBP-1 in male SAC-WT (white bars) (n = 9) and SAC-KO (black bars) (n = 8) mice and female SAC-WT (white bars) (n = 11) and SAC-KO (black bars) (n = 13) mice. Values are presented as the means ± SEM; *, p < 0.05.

The expression levels of *Igf1* mRNA and *Igfbp1* mRNA were measured by qRT-PCR. As shown in Figure 4A and B, males and females demonstrated a significant downregulation of *Igf1* by approximately 80% and 60%, respectively. *Igfbp1* mRNA was upregulated by approximately 3-fold in males and 8-fold in females (Figure 4C, D). Neither *Igfbp2* nor *Igfbp3* changed significantly (Figure 4C, D). Determination of the levels of IGF-I protein in serum revealed strong downregulation in males and females (Figure 4E, F), whereas the upregulation of IGFBP-1 was significant only in female mice.

Completely similar results were obtained with a second transgenic mouse model, the SLC-KO mice (Additional file 4: Figure S4). Deletion of *Smo* in these mice is inducible by transient exposure to Doxycycline at any desired age of the animals. When the *Smo* knockout was induced at 8 weeks of age, the SLC-KO mice show an immediate reduction in weight gain during the next 5 weeks, the same significant alterations of *Gli3, Igf1* and *Igfbp1* expression levels in isolated hepatocytes, and the corresponding changes in IGF-I and IGFBP-1 serum protein concentrations (Additional file 4: Figure S4). These findings convincingly demonstrate that the consequences of hepatocellular deletion of *Smo* are independent upon the specific mechanisms for conditional expression of Cre recombinase and other characteristics of the two types of transgenic mice.

### Gli3 is a transcriptional activator of *Igf1*

**Figure 5:**
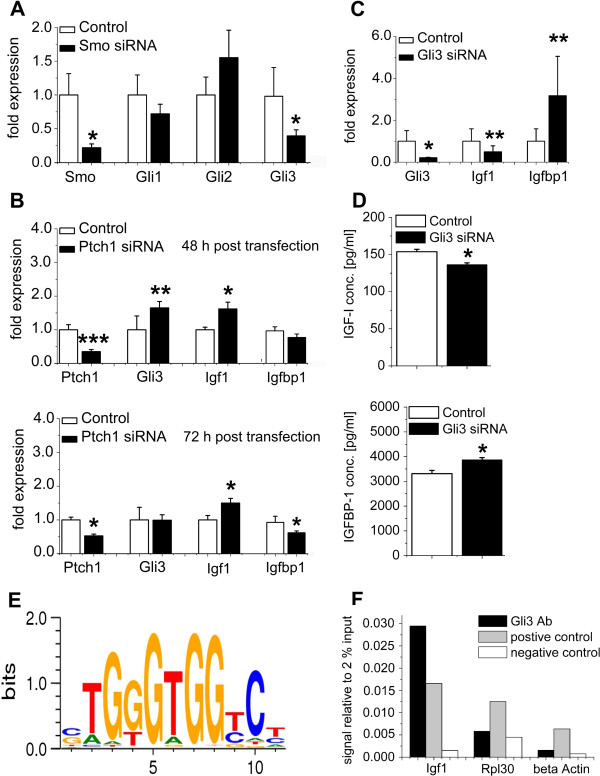
**RNA interference, ELISA and ChIP experiments. (A)**: Relative expression of *Smo, Gli1, Gli2 and Gli3* determined by qRT-PCR analyses in cultured hepatocytes of male C57BL/6-N mice in response to transfection of *Smo* siRNA (black bars) (n = 8) compared to nonsense transfection (white bars) (n = 8) after 48 h of incubation. Values are presented as relative means ± SEM; *, p < 0.05. **(B)**: Relative expression of *Ptch1*, *Gli3*, *Igf1* and *Igfbp1* determined by qRT-PCR analyses in cultured hepatocytes of male C57BL/6-N mice in response to transfection with *Ptch1* siRNA (black bars) compared to nonsense transfection (white bars) after 48 h (n = 10-12) and 72 h (n = 5-6) of incubation. Values are presented as relative means ± SEM; *, p < 0.05; **, p < 0.01; ***, p < 0.001. **(C)**: Relative expression of *Gli3* determined by qRT-PCR analyses in cultured hepatocytes of male C57BL/6-N mice in response to transfection of *Gli3* siRNA (black bars) (n = 11) compared to nonsense transfection (white bars) (n = 11) after 72 h of incubation and relative expression of *Igf1* and *Igfbp1* in C57BL/6 N-hepatocytes after 72 h of incubation with *Gli3* siRNA (black bars) (n = 8) compared to nonsense transfection (white bars) (n = 8). Values are presented as relative means ± SEM; *, p < 0.05; **, p < 0.01. **(D)**: Supernatant analyses of secreted IGF-I and IGFBP-1 in cultured hepatocytes of male C57BL/6-N mice in response to transfection of *Gli3* siRNA (black bars) (n = 6) compared to nonsense transfection (white bars) (n = 6) after 72 h of incubation. Values are presented as the means ± SEM; *, p < 0.05. **(E)**: GLI3 binding site at -405 bp of the *Igf1* promoter according to MotifMap. **(F)**: qRT-PCR analyses of precipitated DNA. DNA immunoprecipitated with GLI3 Antibody (black), Histone H3 Antibody as positive control (grey), and IgG as a negative control (white) calculated relative to input.

To gain insight into the mechanism through which Hh signaling might control the expression of *Igf1* and *Igfbp1*, RNA interference experiments were performed in cultured hepatocytes from C75BL/6-N mice. Because *Gli1* and *Gli3* were significantly down-regulated in SAC-KO mice, we wanted to know which Gli-factor is the predominant one responding immediately to the loss of *Smo*. As shown in Figure 5A, downregulation of *Smo* by *Smo* siRNA resulted in the significant decrease of *Gli3* mRNA level within 48 h, while that of *Gli1* was decreased only by trend at this time. Therefore, we focused on *GLI3* in subsequent *in vitro* experiments. First, we asked whether the downregulation of *Gli3* might be sufficient to account for the observed changes in the expression of *Igf1*. As expected, transfection of cultured hepatocytes with *Gli3* siRNA depleted the *Gli3* mRNA level by 80% (Figure 5C). The decrease in *Gli3* expression was paralleled by a significant decrease in *Igf1* mRNA (Figure 5C), which was in perfect agreement with the results obtained in SAC-KO mice. Moreover, the knockdown lead to a significant decrease in IGF-I protein determined by ELISA in the culture medium after 72 h (Figure 5D).

Second, we were interested in whether the upregulation of Hh signaling causes the opposite regulatory response by the *Igf1* gene. In line with other studies [19], the siRNA-mediated downregulation of *Ptch1* gene expression was chosen to activate Hh signaling. Hepatocyte transfection with *Ptch1* siRNA resulted in significant and selective downregulation of *Ptch1* expression and significant upregulation of *Gli3* after 48 h (Figure 5B), reflecting the known activation of the Hh pathway through the loss of *Ptch1*[26]. In addition, upregulation of the pathway was also indicated by the upregulation of *Gli1*, *Gli2* and *Hhip1* (Additional file 5: Figure S5B,D). Other Hh components (e.g. *Smo*, *Ihh*, *Shh*, *Fu* and *Sufu*) showed no changes in expression, when the pathway was activated via *Ptch1* siRNA (Additional file 5: Figure S5A,C). When *Igf1* expression was evaluated in these experiments, a significant upregulation of *Igf1* mRNA levels was observed that persisted till 72 h (Figure 5B). Together, these results suggest that *Igf1* is a direct transcriptional target of the activator function of GLI3. Data mining by MotifMap [27] provides three different possible binding sites for *Gli3* within the range of -5000 bp upstream to 5000 bp downstream of the transcription start site (Additional file 6: Table S5). One of these located at -405 bp upstream with the sequence TGTGTGGTC shows the most promising features (Figure 5E) and, thus, was selected for a chromatin immunoprecipitation (ChIP) analysis. As shown in Figure 5F GLI3 binding to the selected sequence could definitively be proven by the ChIP assay. Both, positive and negative controls showed the expected results.

Collectively, our results from hepatocytes *ex vivo* (SAC-KO mice) and *in vitro* clearly show that the activity of the Hh signaling pathway in the liver is a major determinant of circulating IGF-I levels. To date, hepatic *Igf1* expression is considered to be primarily regulated by growth hormone via STAT5 [28,29]. Interestingly, in our SAC mice no correlation was found between the serum levels of growth hormone (GH) and IGF-I (correlation coefficient = 0.2488; p = 0.4124) indicating that GH is not the dominant regulator of IGF-I serum levels. Whether Hh signaling acts independently of or interferes with GH signaling in hepatocytes remains to be established.

### Gli3 represses the expression of *Igfbp1* but does not affect that of *Igfbp2* and *3*

In contrast to *Igf1*, the mRNA level of *Igfbp1* was upregulated in livers of SAC-KO mice (Figure 4C). Therefore, we wanted to know whether siRNA-mediated knockdown of *Gli3* in cultured hepatocytes also results in an inverse response of *Igfbp1* mRNA *in vitro*. Indeed, an inverse picture was found, characterized by the selective upregulation of *Igfbp1* expression in response to *Gli3* knockdown (Figure 5C). Likewise, the concentration of IGFBP-1 protein secreted into the culture medium increased significantly after a 72 h cultivation period (Figure 5D).

Conversely, activation of the Hh pathway by *Ptch1* knockdown resulted in significant downregulation of *Igfbp1* mRNA within 72 h (Figure 5B). Apparently, the response here is slightly delayed, because at 48 h after *Ptch1* knockdown the effect is seen only by trend. Again, there is a nice correspondence to the presence of several binding sites for GLI3 in the *Igfbp1* promoter region (Additional file 6: Table S5). In contrast, modulation of Hh signaling *in vivo* or of *Gli3* activity *in vitro* did not show any response at all on the mRNA level of *Igfbp2* and *3*, for which no GLI3 binding sites could be identified using MotifMap (data not shown).

## Discussion

The results obtained with newly generated transgenic mice with hepatocyte-specific conditional knockout of *Smo* reported herein replenished by RNAi experiments using primary hepatocyte cultures demonstrate for the first time that Hedgehog signaling is active in healthy mature liver parenchymal cells and has considerable impact for liver function, and – via modulating liver-derived endocrine factors – for the entire organism. Even though we agree with published data [30] that the levels of Hh signaling components are low in hepatocytes, complete ablation of *Smo* at early or late stages of development as in both, the SAC-KO and the SLC-KO mice, leads to several characteristic phenotypic alterations: a) an obvious growth retardation resulting in lower body size and weight gain, b) a reduced liver/body weight ratio, and c) altered levels of production of IGFI and IGFBP-1 by the liver. Furthermore, changes in the expression of some Hh components, particularly of *Patched1*, were noted indicating the expected response to the interruption of the Hh pathway. Likewise, the Gli transcription factors *Gli1* and *Gli3* were significantly downregulated *in vivo* at the mRNA level. The fact that knockdown of *Smo* in vitro resulted only in significant downregulation of *Gli3* mRNA is compatible with the well-known finding that *Gli1* is a target gene of GLI3 [31]. These findings provide a first proof of principle for our recent hypothesis concerning the existence of a measurable activity of Hh signaling in mature hepatocytes [18].

Since Hh signaling in hepatocytes is currently considered to be mainly associated with hepatocellular damage and resulting regenerative phenomena [17], we carefully investigated molecular markers for damage and precursors of hepatocytes, but found no overt signs of cell death, of activation or relative shifts of non-parenchymal cell populations, and of other changes of liver architecture in the transgenic mice. Thus, there is certainly an effective Hh signaling that is not associated with hepatocyte toxicity and/or replacement. Instead, zonation of the liver seemed to be affected which is of utmost importance for liver function [32,33]. For example, the production of IHH was found to be restricted to pericentral hepatocytes in control livers and was downregulated in SAC-KO livers to an even smaller pericentral area (c.f. Figure 3E). These findings are in line with reports that IHH is a target gene of Wnt/beta-catenin signaling [34] and indicate that the knockout of *Smo* affects Wnt/beta-catenin signaling as predicted recently [18]. Details of this crosstalk between the two morphogen signaling pathways are currently investigated.

Another major result of this study is the unequivocal evidence that hepatocellular Hh signaling is involved in controlling the IGF-I axis in the mouse. The observed decreased levels of *Igf1* mRNA in hepatocytes and of IGF-I protein in serum are in line with the fact that liver-derived IGF-I is the principle source of this hormone in blood [35]. Mechanistically, our results from RNAi experiments on cultured hepatocytes *in vitro* encompassing downregulation of Hh signaling by Gli siRNAs and, conversely, upregulation by *Ptch1* siRNA strongly suggest that Hh signaling acts via the activator function of GLI3 in the transcriptional control of *Igf1*. Indeed, two active GLI3 binding sites in the promoter region of the *Igf1* gene have already been identified by McMahon and co-workers [36], while data mining by MotifMap [27] provides even three different possible binding sites within the range of -5000 bp upstream to 5000 bp downstream of the transcription start site (Additional file 6: Table S5). The sequence TGTGTGGTC located at -405 bp upstream of the transcription start site which we selected for a ChIP analysis nicely fits with the consensus sequence for GLI3 binding sites (Figure 5E). Binding of GLI3 to this sequence could definitively be proven by the ChIP assay, while both positive and negative controls showed the expected results (Figure 5F).

The contribution of Hh signaling to *Igf1* transcription in liver and to the control of serum levels of IGF-I is a novel finding reported for the first time. Hitherto, transcription of the *Igf1* gene was thought to be mainly controlled by growth hormone mediated by dispersed STAT5b-binding elements [28,29]. Our finding that in the SAC mice the IGF-I serum level does not correlate with growth hormone levels argues against an exclusive role of growth hormone. Thus, it seems an important challenge for endocrinologists to elucidate the interplay between these two regulatory mechanisms influencing IGF-I levels. In particular, it remains to be determined whether regulation of *Igf1* expression by GLI3 affects its regulation by STAT5.

Further, we provide strong evidence that IGFBP-1 which is also mainly produced in the liver [37,38] is also influenced by hepatocyte Hh signaling via a *Gli3*-mediated mechanism. So far, IGFBP-1 is known to be inversely regulated by insulin [39]. Though plasma insulin levels are lower in SAC mice of both genders and, thus might contribute to the changes in IGFBP-1 *in vivo*, our *in vitro* results clearly show that the influence by Hh is independent of insulin. Since IGF-I and IGFBP-1 were found to be inversely regulated by *Smo* knockdown it is tempting to speculate that in the case of IGFBP-1 GLI3 may act in a repressive manner, since GLI binding sites were predicted in the promoter region of the IGFBP-1 gene (Additional file 6: Table S5). This would be compatible with the known fact that GLI3 has both repression and activation domains depending on the activation status [8]. Thus, GLI3 may suppress *Igfbp1* expression following knockdown of *Gli3* when GLI3 is truncated to the repressor form. However, the situation is not as clear as for the activating role in the case of IGF-I for two reasons: a) there are no suitable antibodies available that recognize solely the truncated repressor form of GLI3, and b) at present it cannot be excluded that GLI3 acts via repression of an as yet unknown activator of *Igfbp1* expression (unpublished observation). Nonetheless, our results suggest that GLI3 is an important mediator in the regulation of *Igf1* and *Igfbp1* expression by Hh signaling in mouse hepatocytes.

Since IGF-I is long known as an important growth factor with high impact on skeletal growth and body size [40], it is tempting to ask whether the observed changes in the IGF-I axis found in the SAC-KO mice are responsible for the observed changes in body size and weight gain of these mice. Although there is general agreement that liver-derived IGF-I is the principle source of this hormone in blood, its role for postnatal growth in mice remains controversial [35,41,42]. Interestingly, the data obtained in this study shows a clear correlation between IGF-I levels in serum and body weight for male (correlation coefficient = 0.8422; p = 0.0003) and for female (correlation coefficient = 0.6814; p = 0.0026) mice (Additional file 7: Figure S6A,B). Likewise, the correlation between IGFBP-1 and body weight is highly significant also in female mice (correlation coefficient = -0.4834; p = 0.0493) confirming earlier results obtained with mice overexpressing IGFBP-1 [43]. To our surprise, however, there is no such correlation for GH in both genders (correlation coefficient = 0.3356; p = 0.2623 in males and correlation coefficient = 0.2747; p = 0.4136 in females) suggesting that the dominant factors for determining body weight and size in our mice are members of the IGF axis rather than GH.

Apart from contributing to body size, the physiological consequences of the modulation of the IGF axis in SAC-KO mice may be manifold. Liver-derived IGF-I was found to be important for normal carbohydrate and lipid metabolism [41]. Particularly, IGF-I contributes to maintenance of normal glucose homeostasis and is necessary for normal insulin sensitivity [44]. Likewise, IGFBP-1 is recognized as an important regulator of glucose levels and a potential marker for the metabolic syndrome [45]. Our findings that plasma insulin levels are decreased in SAC-KO mice, while refeed glucose levels were slightly decreased rather than increased generally reflect an important influence of hepatic Hh signaling on glucose homeostasis mediated, at least in part, by modulation of the IGF axis. However, since the effects of IGF-I and IGFBP-1 are opposite in nature [44,45], it is hard to draw conclusions about the individual contribution of these factors in the context of our transgenic mice.

Another important consequence of the altered hepatic Hh signaling may concern the reproductive capacity. Both, IGF-I and IGFBP-1 were found to interfere with reproductive functions particularly in females [46,47]. Most interestingly, low IGF-I serum levels and/or upregulated levels of IGFBP-1 are associated with considerably impaired fertilization or implantation [37]. Indeed female SAC-KO mice show a strongly reduced reproductive capacity in line with the effects ascribed to the alterations in the IGF-I axis rather than with GH. The anatomical and physiological aspects of this effect are currently investigated in detail.

## Conclusions

In conclusion, the results of this study, obtained using different transgenic mice with conditional hepatic knockout of *Smo*, demonstrate for the first time that Hh signaling is active in healthy mature hepatocytes and has considerable impact on endocrine liver function. The consequences are manifold and comprise phenomena as different as body size and weight, glucose metabolism and homeostasis or female fertility.

## Materials and methods

### Generation of transgenic SAC mice

Transgenic mice with hepatocyte-specific knockout of *Smoothend* (*Smo*) were obtained by crossing *Smo*^flox/flox^ and Alfp-Cre lines (Additional file 1: Figure S1A-C). After establishing the animals the general breading protocol was as follows: male mice: *Smo*^flox/flox^ + Cre-recombinase mated with female *Smo*^flox/flox^ - Cre recombinase. In agreement with the results of Kellendonk et al. [20], Cre-recombinase could be detected by immunohistochemistry in all hepatocytes and cholangiocytes (Additional file 1: Figure S1D).

Genotypes of the transgenic mice were identified by PCR using DNA from tailtips. After birth mice were screened for expressing the *Smo* wildtyp (*Smo* WT), the *Smo* floxed (*Smo* flx.) allele and the Cre-recombinase by PCR. All primers are listed in Additional file 6: Table S1.

### Generation of transgenic SLC mice

Triple transgenic mice allowing conditional hepatocyte-specific ablation of *Smo* in response to exposure to Doxycycline (abbreviated SLC mice) were generated by crossing the *Smo*^tm2Amc^/J mice (Jackson Laboratories) possessing *loxP* sites on either side of exon 1 of the *Smo* gene [48] with double transgenic LC-1/rTA^LAP^ -1 mice [49] (generously provided by Hermann Bujard). For inducing ablation of *Smo*, SLC mice were grown up for 8 weeks under normal conditions. Then, expression of Cre-recombinase was induced by adding Doxycycline hydrochloride (2 mg/ml) to the drinking water for a period of 10 days.

### Maintenance of mice and feeding

Mice were maintained according to the German guidelines and local regulations for the care and safe use of experimental animals in a pathogen-free facility in a 12:12 h LD cycle (permission numbers: TVV11/08; N05/12; A19/11). Animals had free access to regular chow (ssniff® M-Z V1124-0 composed of 22.0% protein, 50.1% carbohydrate, 4.5% fat; usable energy: 13.7 kJ/g; ssniff® Spezialdiäten GmbH, Soest, Germany) and tap water throughout life. Before sacrifice (between 9 and 11 am), mice were starved for 24 h and re-fed with regular chow for 12 h, in order to obtain a synchronized feeding state.

### Measurements in blood samples and cell culture-supernatant

Blood samples were taken from the beating heart of anesthesized mice. Serum activities of ASAT, ALAT, and GLDH were measured with an automated analyser (Roche modular) using standardized assays (Roche, Mannheim, Germany). Blood glucose levels were determined using a Blood Glucose Meter (Freestyle Mini, Abbot, Wiesbaden).

Insulin was detected in serum with an Elisa- Kit from DRG Instruments (Marburg, Germany) (EIA 3439). IGF-I, IGFBP-1 and GH, were detected in serum with Elisa-Kits from Mediagnost (Reutlingen, Germany and Cell Applications (San Diego, USA).

To determine IGF-I and IGFBP-I in cell culture supernatant we used Elisa-Kits from Boster Biological Technology (Fremont, CA, USA).

### Isolation and cultivation of primary hepatocytes

Primary hepatocytes from SAC mice were isolated by collagenase perfusion of the liver as described [50]. Cell suspension was carefully cleared from non-parenchymal cells by differential centrifugation. Finally, hepatocytes were suspended in Williams Medium E containing 10% fetal calf serum and further additions as described [50,51], and were plated onto 12-well plates precoated with collagen type 1. After 2 h, serum-free medium was used throughout cultivation.

To give an overview of the purity of our primary hepatocyte isolation we looked for the expression of typical markers of mature hepatocytes like *Arg1* (*Arginase 1*), *Gck* (*Glucokinase*), *Gls2* (G*lutaminase 2* (liver, mitochondrial)), *Ldha* (*lactate dehydrogenase A chain*), *Krt18* (*Keratin 18*) and *Krt8* (*Keratin 8*). For the non-parenchymal cell population (e.g. hepatic stellate cells, vascular smooth muscle cells kupffer cells and cholangiocytes) we analyzed *Gfap* (*glial fibrillary acidic protein*), *Acta2* (*actin, alpha 2, smooth muscle, aorta*), *Emr1* (*EGF-like module containing, mucin-like, hormone receptor-like sequence 1*), and *Krt19* (*Keratin 19*). All genes were analyzed in SAC-WT and SAC-KO material (Additional file 3: Figure S3A,B,C,D).

### RNA preparation and quantitative real-time PCR (qRT-PCR)

Total RNA from hepatocytes, liver tissue and other organs was extracted using TRIzol (peqlab, Erlangen, Germay). RNA was reverse transcribed using oligo(dt) primers and Proto Script M-MuLV First Strand cDNA Synthesis Kit (New England Biolabs, Frankfurt am Main, Germany). The resulting cDNA samples were then quantified for each test gene using target gene-specific primers designed using the online tools Universal Probe Library software, Perl Primer and Primer 3. Primers are listed in Additional file 6: Table S2. The levels of all mRNA transcripts were determined in duplicate by qRT-PCR using the Light Cycler® 2.0 Instrument and the Light Cycler® Fast Start DNA Master^plus^ SYBR Green I (Roche, Mannheim, Germany). Using the standard curve method, the absolute quantification of specific PCR products for each primer set was performed. For normalization, *β-actin* was amplified from each sample.

### RNA-interference

*Smo*-, *Ptch1*- and *Gli3*-specific siRNAs and respective nonsense control siRNAs were purchased from Invitrogen, Darmstadt, Germany. Freshly isolated hepatocytes were seeded at a density of 100000 cells per well of 12-well plates in normal culture medium containing 10% FCS. After 4 h, serum-free medium was used and chemically synthesized siRNAs (10 nmol for *Smo* and *Ptch1*, 25 nmol for *Gli3*) were transfected with Interferin from peqlab, Erlangen, Germany, according to the manufacturer’s instructions. Sequences for siRNA primers are listen in Additional file 6: Table S3. Twenty-four hours after transfection, the medium was changed and fresh medium without siRNA was added. Changes in gene expression were analysed by qRT-PCR at 48 h and 72 h post-transfection.

### Chromatin Immunoprecipitation (ChIP)

To determine the binding of the transcription factor GLI3 to the *Igf1* promoter region we used the SimpleChIP Plus Enzymatic Chromatin IP Kit (Agarose Beads) (Cell Signaling, Erlangen, Germany) according to the manufacturer’s instructions. Freshly isolated hepatocytes from three male C57BL/6 mice were pooled, washed and cross-linked with 37% formaldehyde. As positive experimental control the Histone H3 (D2B12) XP® Rabbit mAb (#2729) was used whereas normal Rabbit IgG served as negative control. The cross-linked GLI3 DNA complex was precipitated with the goat anti mouse GLI3 antibody (20 μg) (R&D Wiesbaden-Nordenstadt, Germany). The quantification analysis was performed using qRT-PCR with 5 μl of each DNA sample and specific primers listed in Additional file 6: Table S4. Primer pairs for the putative GLI3 binding site in the *Igf1* promoter region were designed using Primer-BLAST of NCBI [52]. The range of interest in the promoter region (~ -405 bp) was obtained by the MotifMap [27] analysis. The applicability of the primer pair was tested using the UCSC *In silico* PCR analysis (http://genome.csdb.cn/cgi-bin/hgPcr). The desired *Igf1* amplicon resulted in a 283 bp fragment. The primers for *β–actin* were used to analyse the unspecific DNA-Antibody-binding. *Rpl30* primers (included in the SimpleChIP Plus Enzymatic Chromatin IP Kit) were used as a positive control for the histone H3 antibody precipitation.

### Immunohistochemistry

Immunohistochemistry on paraffin sections (3 μm) was performed similarly as described [53]. After dewaxing and rehydration sections for Cre-recombinase, IHH, GLI3 and PKM2 staining were microwaved (3 × 5 min) in citrate buffer (0.01 M sodium citrate, pH 6.0) for antigen retrieval. For GFAP staining the slides were incubated for 4 min with 0.1% Proteinase K and 0.1% Calcium for antigen retrieval. Endogenous peroxidase was destroyed by incubation with 3% hydrogen peroxide in TBS (0.01 M Tris-base, 0.9% NaCl, pH 7.6) for 10 min. To block unspecific binding, slides for Cre-Recombinase, GFAP, IHH and GLI3 staining were incubated for 30 min in 5% goat serum (Sigma, München, Germany) and slides for PKM2 staining were incubated for 30 min in 10% horse serum (Sigma, München, Germany). Primary antibodies were rabbit anti Cre-recombinase (1:4000; Abcam, Cambridge, UK), rabbit anti Indian Hedgehog (1:250, Abcam, Cambridge, UK), rabbit anti GLI3 (1:250, GeneTex, Irvine, USA), rabbit anti Gfap (1:1000, Dako, Hamburg, Germany) rabbit anti Pyruvate Kinase muscle (1:500, Biomol, Hamburg, Germany). To visualize IHH and GLI3 proteins the Dako REAL™ EnVision™ Detection System, Peroxidase/DAB+,Rabbit Mouse (Dako, Hamburg, Germany) was used according to the manufacturer’s protocol. Secondary antibody for Cre Recombinase, PKM2 and GFAP staining was biotinylated goat anti-rabbit IgG (Millipore, Schwalbach, Germany) used together with Extravidine (Sigma, München, Germany). Diaminobenzidine and Aminotriazole were used for POD staining as described [53]. Sections were counterstaining with hematoxylin.

### Statistical analysis

Values are expressed as means ± standard deviation of the mean (SEM) and sample size (n) is given in each figure ore table. Statistical evaluation was performed using the Mann–Whitney-*U*-Test for fresh tissue and hepatocytes and the wilcoxon matched pairs test for the siRNA experiments in cultured hepatocytes. The null hypothesis was rejected at the p < 0.05 (*), p < 0.01 (**) and p < 0.001 (***) levels. The Pearson correlation coefficients and the related p-values for Pearson’s correlation were calculated using the Statistics toolbox of MATLAB (The MathWorks Inc., Natick, MA, USA).

### Availability of supporting data

The data sets supporting the results of this article are included within the article (and its additional files).

## Abbreviations

Acta2: *actin, alpha 2, smooth muscle, aorta*; ALAT: Alanine aminotransferase; ASAT: Aspartate aminotransferase; Arg1: *arginase 1*; BBLS: Bayesian branch length score; BLS: Branch length score; Boc: *brother of Cdo*; Cd34: *cd34 antigen*; Cdo: *cell adhesion molecule-related/downregulated by oncogenes*; Dhh: *dessert hedgehog*; Emr1: EGF-like module containing, mucin-like, hormone receptor-like sequence 1; FDR: False discovery rate; Gck: *glucokinase*; GLDH: Glutamate dehydrogenase; Gls2: *glutaminase 2*; Gfap: *glial fibrillary acidic protein*; Gli1: *glioma-associated oncogene family zinc finger 1*; Gli2: *glioma-associated oncogene family zinc finger 2*; Gli3: *glioma-associated oncogene family zinc finger 3*; Hh: Hedgehog; IGFBP-1: Insulin-like growth factor binding protein 1; IGFBP-2: Insulin-like growth factor binding protein 2; IGFBP-3: Insulin-like growth factor binding protein 3; IGF-I: Insulin-like growth factor 1; Ihh: *Indian hedgehog*; Krt8: *keratin8*; Krt18: *keratin18*; Krt19: *keratin 19*; Ldha: *lactate dehydrogenase A*; NLOD: Normalized log-odds score; Pkm2: *pyruvate kinase, muscle*; Ptch1: *patched1*; Ptch2: *patched2*; Shh: *sonic hedgehog*; Smo: *smoothened*.

## Competing interests

GR is listed as the inventor on a patent application filed by the University of Leipzig (PCT/EP2013/051425, Therapeutic use of activators of zinc finger protein GLI3).

## Authors’ contributions

MSM designed and conducted most experiments, analyzed the data and wrote the manuscript. AS, BJ, KJ, AK and ME designed and conducted the experiments and analyzed the data. SHW analyzed the data. GR conceived the project, analyzed the data, and wrote and corrected the manuscript. All authors read and approved the final version of the manuscript.

## Supplementary Material

Additional file 1: Figure S1 Generation of the transgenic mouse model with hepatocyte-specific deletion of *Smoothened*. **(A)**: Structure of the Alfp-Cre vector construct. **(B)**: The Smo locus with loxP sites on either side of exon 1in the absence of Cre-recombinase activity. **(C)**: The floxed Smo locus lacking exon 1 in the presence of Cre-recombinase activity. **(D)**: Immunohistochemical detection of Cre-recombinase in liver sections of SAC-WT and SAC-KO mice. Brown colour indicating Cre-recombinase is present in hepatocyte and cholangiocyte nuclei (strong staining) and cytoplasm (weak staining). Bar: 50 μm. **(E)**: PCR analysis, using DNA extracted from liver tissue of SAC-WT and SAC-KO mice yields a 600-bp amplicon of wild-type *Smo* in different tissues SAC-WT (black bars) (n = 7-20) and SAC-KO (white bars) (n = 7-20) mice determined by qRT-PCR. Significant decrease of *Smo* mRNA relative to *β-actin* is detected only in liver. Values are presented as means ± SEM; *, p<0.05. Click here for file

Additional file 2: Figure S2 Phenotypic features induced by hepatocyte-specific ablation of *Smoothened* in transgenic SAC mice. Photographs of **(A)**: male SAC wildtype SAC-WT (*Smo*^+/+^), heterozygous SAC-KO (*Smo*^+/-^) and homozygous SAC-KO (*Smo*^-/-^) mouse. **(B)**: Comparison of body weight of 4, 8 and 12 weeks old male SAC-WT (*Smo*^+/+^) (white bars) (n = 4-9), heterozygous SAC-KO (*Smo*^+/-^) (gray bars) (n = 8-9) and homozygous SAC-KO (*Smo*^-/-^) (black bars) (n = 8-21) mice. Values are presented as the means ± SEM; *, p<0.05; **, p<0.01; ***, p<0,001. Click here for file

Additional file 3: Figure S3 Expression of markers for hepatocytes and non-parenchymal cells in liver sections and isolated hepatocytes from male transgenic SAC mice. qRT-PCR analyses of the expression of markers *Arg1 Gck, Gls2, Ldha, Krt8* and *Krt18* in male **(A)**: SAC-WT liver (white bars) (n = 8) versus SAC-WT hepatocytes (hatched bars) (n = 7); **(B)** SAC-KO liver (white bars) (n = 8) versus SAC-KO hepatocytes (hatched bars) (n = 8). Non-parenchymal cell markers *Acta2, Gfap, Emr1,* and *Krt19* in male; **(C)**: SAC-WT liver (black bars) (n = 8) versus SAC-WT hepatocytes (checkered bars) (n = 8); **(D)**: SAC-KO liver (black bars) (n = 8) versus SAC-KO hepatocytes (chekered bars) (n = 8). *, p<0.05;***, p<0.01; ***,p<0.001. Values are presented as the means ± SEM. Click here for file

Additional file 4: Figure S4 Physiological and molecular effect of hepatocellular deletion of *Smo* in transgenic SLC mice. **(A)**: Comparison of body weight of female SLC-WT mice without Doxycycline (Dox.) (white bars) (n = 17), SLC-WT mice with Dox. (gray bars) (n = 4) and SLC-KO mice with Dox. (black bars) (n = 12) at 5 weeks after deletion of *Smo*. Doxycycline was given at the age of 8 weeks for 10 days to induce the Cre-recombinase expression as described in Materials and methods. **(B)**: Comparison of body weight of female SLC-WT mice (black squares) (n = 8-14) and SLC-KO mice (open circles) (n = 8-10). Deletion of *Smo* was induced at the age of 8 weeks. **(C)**: qRT-PCR analyses of the expression of *Smo*, *Gli1, Gli2* and *Gli3* in isolated hepatocytes from female SLC-WT mice (white bars) (n = 5-15) and SLC-KO mice (black bars) (n = 5-16) at 5 weeks after deletion of *Smo*. **(D)**: qRT-PCR analyses of the expression of *Igf1* and *Igfbp1* in isolated hepatocytes from female SLC-WT mice (white bars) (n = 7) and SLC-KO mice (black bars) (n = 4) at k weeks after deletion of *Smo*. *, p<0.05; **, p<0.01; ***, p<0.001. Values are presented as the means ± SEM. Click here for file

Additional file 5: Figure S5 Response of selected Hh pathway genes to siRNA mediated knockdown of Ptch1. qRT-PCR analyses of **(A)**: *Smo*, *lhh* and *Shh*; **(B)**: *Ptch2* and *Hhip1*, **(C)**: *Fu* and *Sufu* and **(D)**: *Gli1* and *Gli2* in cultured hepatocytes of male C57BL/6N mice in response to transfection with *Ptch1* siRNA (black bars) (n = 7-8) compared to nonsense transfection (white bars) (n = 7-8) after 48 h of incubation. Values are presented as relative means ± SEM. Click here for file

Additional file 6: Table S1 Primers for PCR genotyping of SAC mice. **Table S2:** Primers for qRT-PCR analyses. **Table S3:** Primers for RNA interference experiments. **Table S4:** Primers for Chromatin IP. **Table S5:** Binding sites of Gli factors and Gli factor-dependent transcription factors to the promoter region of *Igf1* and *Igfbp-1* in mouse determined by MotifMap. Click here for file

Additional file 7: Figure S6 Correlation analyses of IGF-I serum concentrations and body weight from male and female transgenic SAC mice. Correlation analyses of IGF-I serum concentrations and body weight from **(A)**: male SAC-WT (black circles) (n = 7) mice and SAC-KO (open circles) (n = 6) mice and **(B)**: female SAC-WT (black circles) (n = 9) mice and SAC-KO (open circles) (n = 8) mice. c = correlation coefficient; p = significance level. Click here for file
