# Supplementary material for: Hepatic Hedgehog signaling contributes to the regulation of IGF1 and IGFBP1 serum levels
Source: Cell Commun Signal. 2014 Feb 18;12:11. doi: 10.1186/1478-811X-12-11 (PMC3946028; doi:10.1186/1478-811X-12-11)
Supplement: Additional file 1: Figure S1 — Generation of the transgenic mouse model with hepatocyte-specific deletion of Smoothened. (A): Structure of the Alfp-Cre vector construct. (B): The Smo locus with loxP sites on either side of exon 1in the absence of Cre-recombinase activity. (C): The floxed Smo locus lacking exon 1 in the presence of Cre-recombinase activity. (D): Immunohistochemical detection of Cre-recombinase in liver sections of SAC-WT and SAC-KO mice. Brown colour indicating Cre-recombinase is present in hepatocyte and cholangiocyte nuclei (strong staining) and cytoplasm (weak staining). Bar: 50 μm. (E): PCR analysis, using DNA extracted from liver tissue of SAC-WT and SAC-KO mice yields a 600-bp amplicon of wild-type Smo in different tissues SAC-WT (black bars) (n = 7-20) and SAC-KO (white bars) (n = 7-20) mice determined by qRT-PCR. Significant decrease of Smo mRNA relative to β-actin is detected only in liver. Values are presented as means ± SEM; *, p<0.05. [file 1478-811X-12-11-S1.pdf]

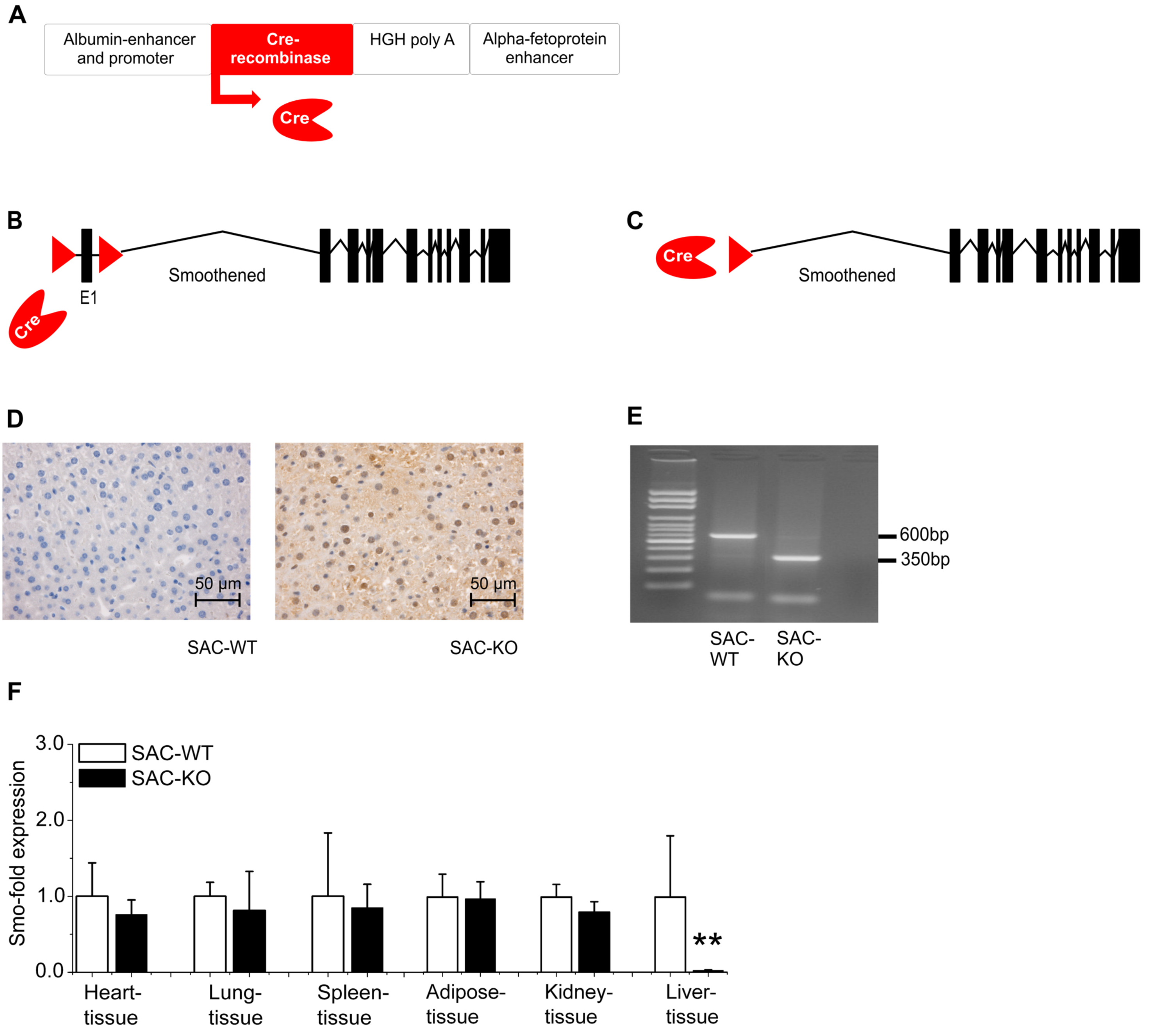

Figure S1: Generation of the transgenic mouse model with hepatocyte-specific deletion of *Smoothened*.
(A): Structure of the Alfp-Cre vector construct. **(B):** The *Smo* locus with loxP sites on either side of exon 1 in the absence of Cre-recombinase activity. **(C):** The floxed *Smo* locus lacking exon 1 in the presence of Cre-recombinase activity. **(D):** Immunohistochemical detection of Cre-recombinase in liver sections of SAC-WT and SAC-KO mice. Brown colour indicating Cre-recombinase is present in hepatocyte and cholangiocyte nuclei (strong staining) and cytoplasm (weak staining). Bar: 50 µm. **(E):** PCR analysis, using DNA extracted from liver tissue of SAC-WT and SAC-KO mice yields a 600-bp amplicon of wild-type *Smo* alleles and a 350-bp amplicon of recombinant *Smo* alleles in the knockout genotype. **(F):** Comparison of the relative expression of *Smo* in different tissues SAC-WT (black bars) (n=7-20) and SAC-KO (white bars) (n=7-20) mice determined by qRT-PCR. Significant decrease of *Smo* mRNA relative to *β-actin* is detected only in liver. Values are presented as means ± SEM; *, p<0.05.
