# Supplementary material for: Hepatic Hedgehog signaling contributes to the regulation of IGF1 and IGFBP1 serum levels
Source: Cell Commun Signal. 2014 Feb 18;12:11. doi: 10.1186/1478-811X-12-11 (PMC3946028; doi:10.1186/1478-811X-12-11)
Supplement: Additional file 2: Figure S2 — Phenotypic features induced by hepatocyte-specific ablation of Smoothened in transgenic SAC mice. Photographs of (A): male SAC wildtype SAC-WT (Smo+/+), heterozygous SAC-KO (Smo+/-) and homozygous SAC-KO (Smo-/-) mouse. (B): Comparison of body weight of 4, 8 and 12 weeks old male SAC-WT (Smo+/+) (white bars) (n = 4-9), heterozygous SAC-KO (Smo+/-) (gray bars) (n = 8-9) and homozygous SAC-KO (Smo-/-) (black bars) (n = 8-21) mice. Values are presented as the means ± SEM; *, p<0.05; **, p<0.01; ***, p<0,001. [file 1478-811X-12-11-S2.pdf]

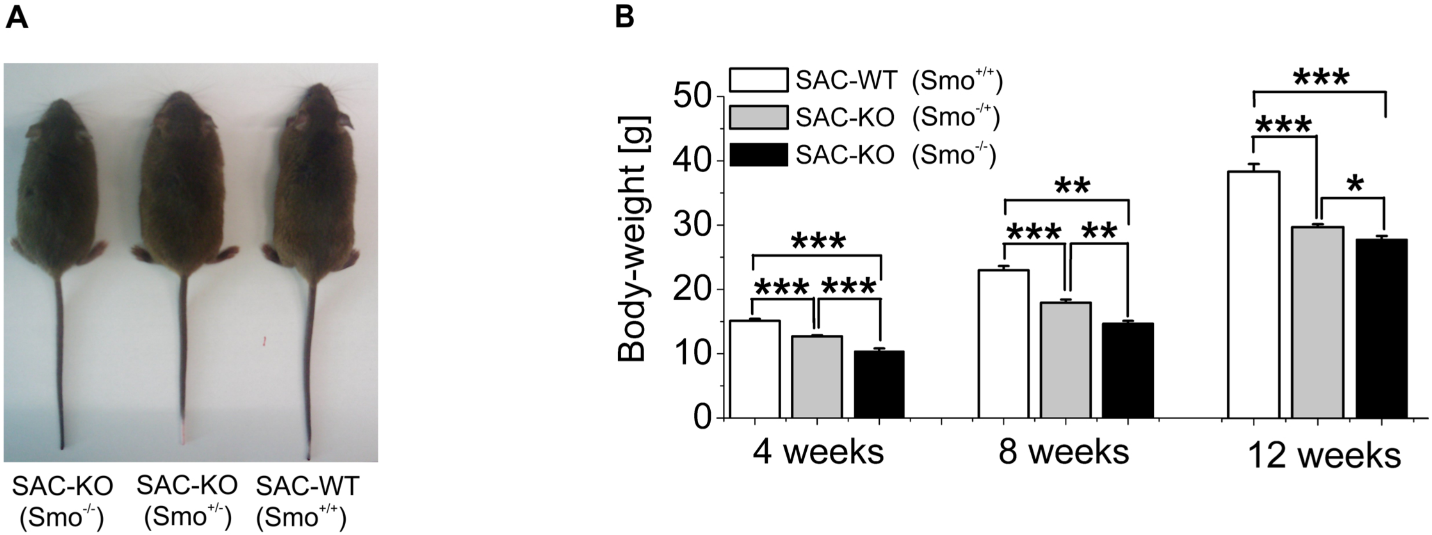

Figure S2: Phenotypic features induced by hepatocyte-specific ablation of *Smoothened* in transgenic SAC mice. Photographs of **(A):** male SAC wildtype SAC-WT (*Smo*$^{+/+}$), heterozygous SAC-KO (*Smo*$^{+/-}$) and homozygous SAC-KO (*Smo*$^{-/-}$) mouse. **(B):** Comparison of body weight of 4, 8 and 12 weeks old male SAC-WT (*Smo*$^{+/+}$) (white bars) (n=4-9), heterozygous SAC-KO (*Smo*$^{+/-}$) (gray bars) (n=8-9) and homozygous SAC-KO (*Smo*$^{-/-}$) (black bars) (n=8-21) mice. Values are presented as the means ± SEM; *, $p<0.05$; **, $p<0.01$; ***, $p<0,001$.
