# Supplementary material for: Hepatic Hedgehog signaling contributes to the regulation of IGF1 and IGFBP1 serum levels
Source: Cell Commun Signal. 2014 Feb 18;12:11. doi: 10.1186/1478-811X-12-11 (PMC3946028; doi:10.1186/1478-811X-12-11)
Supplement: Additional file 3: Figure S3 — Expression of markers for hepatocytes and non-parenchymal cells in liver sections and isolated hepatocytes from male transgenic SAC mice. qRT-PCR analyses of the expression of markers Arg1 Gck, Gls2, Ldha, Krt8 and Krt18 in male (A): SAC-WT liver (white bars) (n = 8) versus SAC-WT hepatocytes (hatched bars) (n = 7); (B) SAC-KO liver (white bars) (n = 8) versus SAC-KO hepatocytes (hatched bars) (n = 8). Non-parenchymal cell markers Acta2, Gfap, Emr1, and Krt19 in male; (C): SAC-WT liver (black bars) (n = 8) versus SAC-WT hepatocytes (checkered bars) (n = 8); (D): SAC-KO liver (black bars) (n = 8) versus SAC-KO hepatocytes (chekered bars) (n = 8). *, p<0.05;***, p<0.01; ***,p<0.001. Values are presented as the means ± SEM. [file 1478-811X-12-11-S3.pdf]

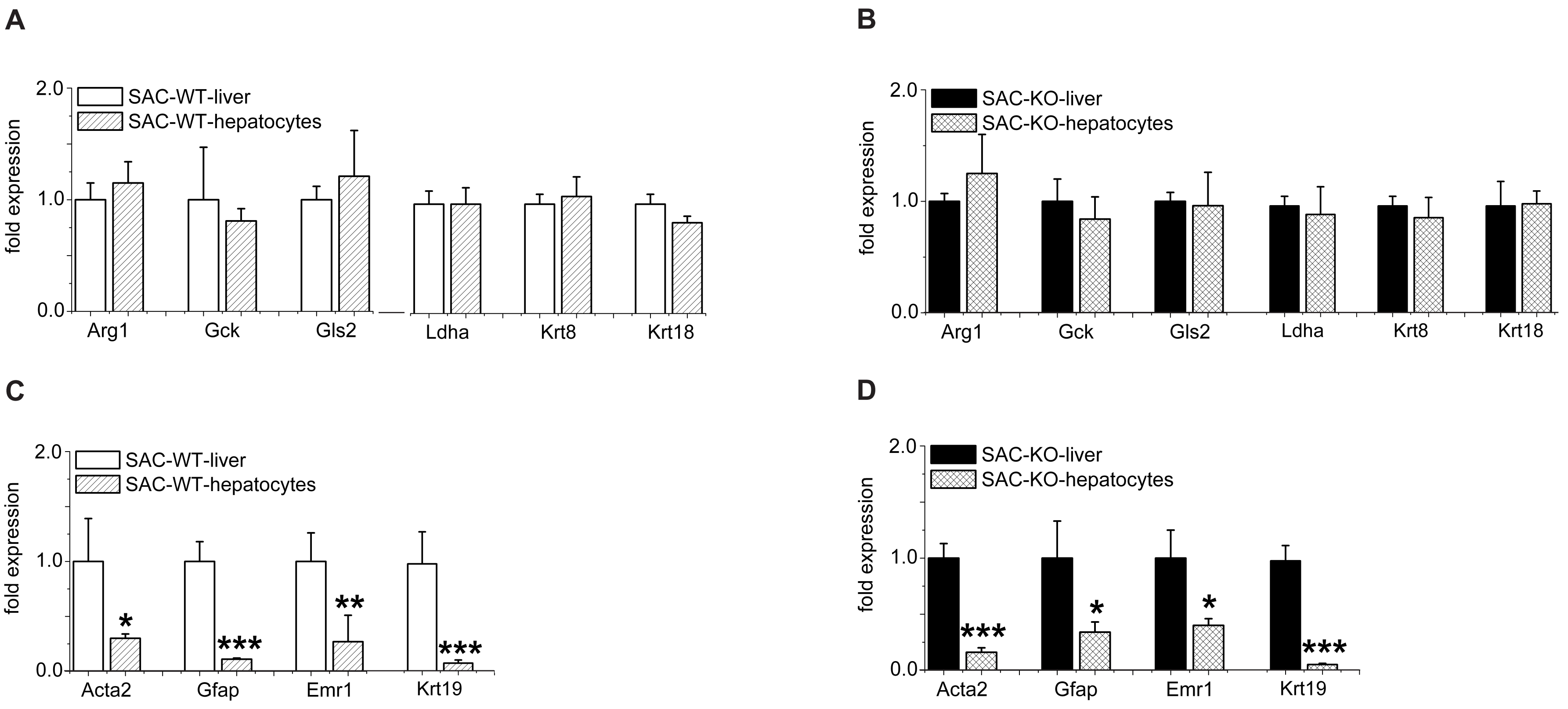

Figure S3: Expression of markers for hepatocytes and non-parenchymal cells in liver sections and isolated hepatocytes from male transgenic SAC mice.

qRT-PCR analyses of the expression of marker genes in liver sections and isolated hepatocytes from **(A,C)** SAC-WT mice and **(B,D)** SAC-KO mice. Mature hepatic markers *Arg1*, *Gck*, *Gls2*, *Ldha*, *Krt8* and *Krt18* in male **(A):** SAC-WT liver (white bars) (n=8) versus SAC-WT hepatocytes (hatched bars) (n=7); **(B):** SAC-KO liver (white bars) (n=8) versus SAC-KO hepatocytes (hatched bars) (n=8). Non-parenchymal cell markers *Acta2*, *Gfap*, *Emr1* and *Krt19* in male; **(C):** SAC-WT liver (black bars) (n=8) versus SAC-WT hepatocytes (checkered bars) (n=8); **(D):** SAC-KO liver (black bars) (n=8) versus SAC-KO hepatocytes (checkered bars) (n=8). *, $p<0.05$; **, $p<0.01$; ***, $p<0.001$. Values are presented as the means ± SEM.
