# Supplementary material for: Hepatic Hedgehog signaling contributes to the regulation of IGF1 and IGFBP1 serum levels
Source: Cell Commun Signal. 2014 Feb 18;12:11. doi: 10.1186/1478-811X-12-11 (PMC3946028; doi:10.1186/1478-811X-12-11)
Supplement: Additional file 4: Figure S4 — Physiological and molecular effect of hepatocellular deletion of Smo in transgenic SLC mice. (A): Comparison of body weight of female SLC-WT mice without Doxycycline (Dox.) (white bars) (n = 17), SLC-WT mice with Dox. (gray bars) (n = 4) and SLC-KO mice with Dox. (black bars) (n = 12) at 5 weeks after deletion of Smo. Doxycycline was given at the age of 8 weeks for 10 days to induce the Cre-recombinase expression as described in Materials and methods. (B): Comparison of body weight of female SLC-WT mice (black squares) (n = 8-14) and SLC-KO mice (open circles) (n = 8-10). Deletion of Smo was induced at the age of 8 weeks. (C): qRT-PCR analyses of the expression of Smo, Gli1, Gli2 and Gli3 in isolated hepatocytes from female SLC-WT mice (white bars) (n = 5-15) and SLC-KO mice (black bars) (n = 5-16) at 5 weeks after deletion of Smo. (D): qRT-PCR analyses of the expression of Igf1 and Igfbp1 in isolated hepatocytes from female SLC-WT mice (white bars) (n = 7) and SLC-KO mice (black bars) (n = 4) at k weeks after deletion of Smo. *, p<0.05; **, p<0.01; ***, p<0.001. Values are presented as the means ± SEM. [file 1478-811X-12-11-S4.pdf]

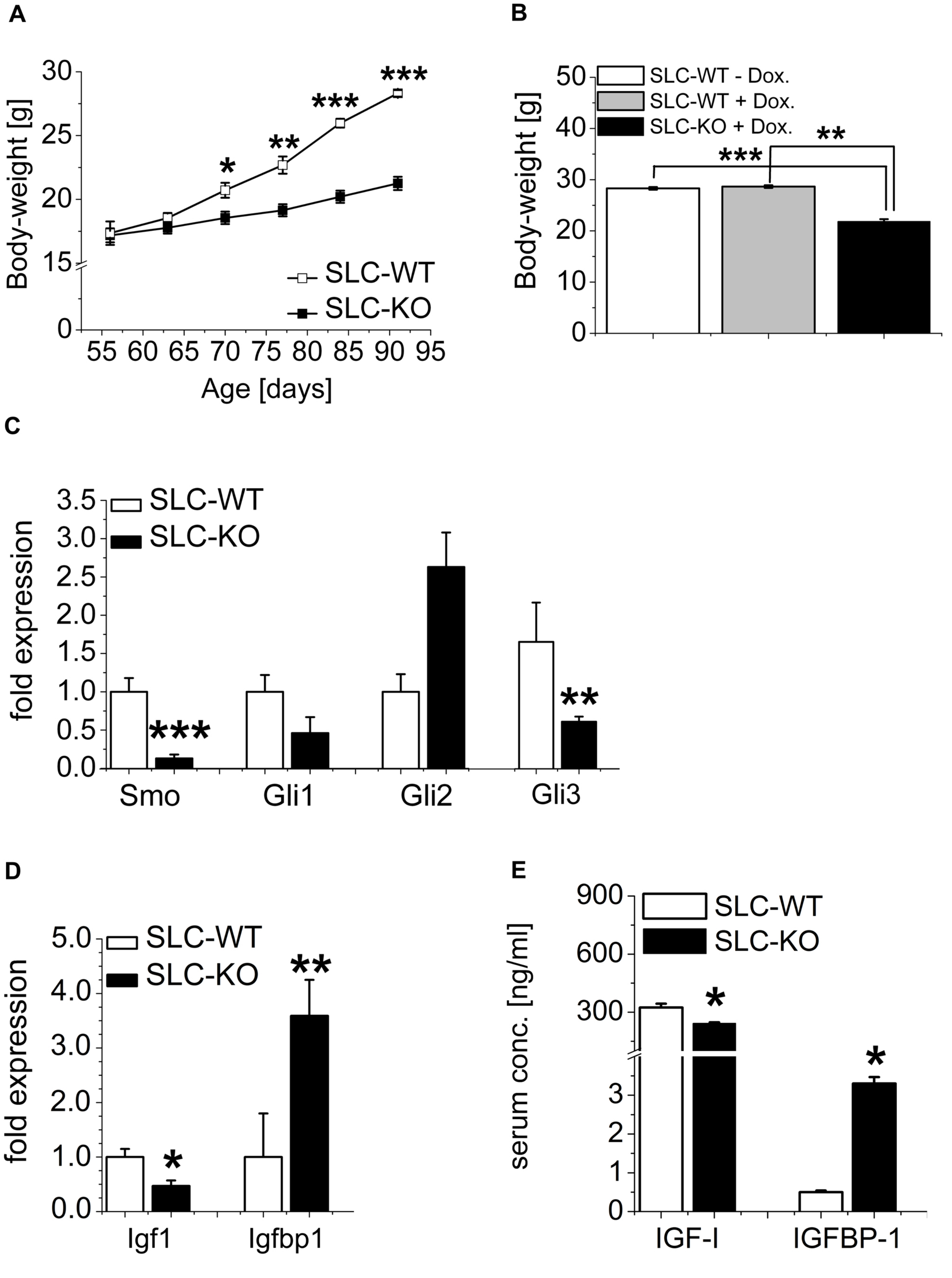

Figure S4: Physiological and molecular effect of hepatocellular deletion of *Smo* in transgenic SLC mice.
(A): Comparison of body weight of female SLC-WT mice without Doxycycline (Dox.) (white bars) (n=17), SLC-WT mice with Dox. (gray bars) (n=4) and SLC-KO mice with Dox. (black bars) (n=12) at 5 weeks after deletion of *Smo*. Doxycycline was given at the age of 8 weeks for 10 days to induce the Cre-recombinase expression as described in materials and methods. **(B):** Comparison of body weight of female SLC-WT mice (black squares) (n=8-14) and SLC-KO mice (open circles) (n=8-10). Deletion of *Smo* was induced at the age of 8 weeks. **(C):** qRT-PCR analyses of the expression of *Smo*, *Gli1*, *Gli2* and *Gli3* in isolated hepatocytes from female SLC-WT mice (white bars) (n=5-15) and SLC-KO mice (black bars) (n=5-16) at 5 weeks after deletion of *Smo*. **(D):** qRT-PCR analyses of the expression of *Igf1* and *Igfbp1* in isolated hepatocytes from female SLC-WT mice (white bars) (n=7) and SLC-KO mice (black bars) (n=9). **(E):** Serum analyses of circulating IGF-I and IGFBP-1 in female SLC-WT mice (white bars) (n=4) and SLC-KO mice (black bars) (n=4) at 5 weeks after deletion of *Smo*. *, $p<0.05$;**, $p<0.01$; ***, $p<0.001$. Values are presented as the means ± SEM.
