# Supplementary material for: Hepatic Hedgehog signaling contributes to the regulation of IGF1 and IGFBP1 serum levels
Source: Cell Commun Signal. 2014 Feb 18;12:11. doi: 10.1186/1478-811X-12-11 (PMC3946028; doi:10.1186/1478-811X-12-11)
Supplement: Additional file 5: Figure S5 — Response of selected Hh pathway genes to siRNA mediated knockdown of Ptch1. qRT-PCR analyses of (A): Smo, lhh and Shh; (B): Ptch2 and Hhip1, (C): Fu and Sufu and (D): Gli1 and Gli2 in cultured hepatocytes of male C57BL/6N mice in response to transfection with Ptch1 siRNA (black bars) (n = 7-8) compared to nonsense transfection (white bars) (n = 7-8) after 48 h of incubation. Values are presented as relative means ± SEM. [file 1478-811X-12-11-S5.pdf]

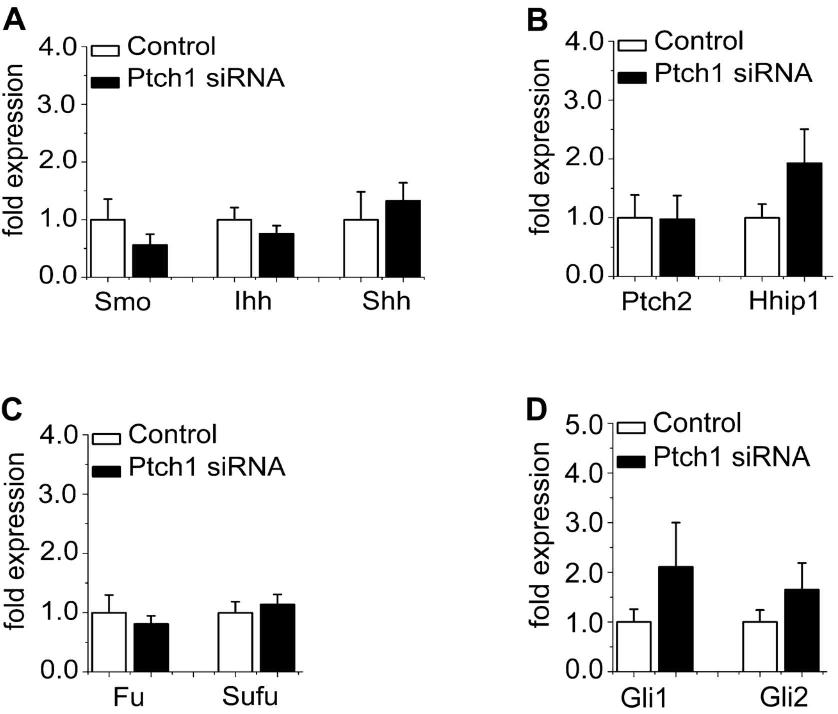

Figure S5: Response of selected Hh pathway genes to siRNA mediated knockdown of *Ptch1*

qRT-PCR analyses of **(A):** *Smo*, *Ihh* and *Shh*; **(B):** *Ptch2* and *Hhip1*, **(C):** *Fu* and *Sufu* and **(D):** *Gli1* and *Gli2* in cultured hepatocytes of male C57BL/6N mice in response to transfection with *Ptch1* siRNA (black bars) (n=7-8) compared to nonsense transfection (white bars) (n=7-8) after 48 h of incubation. Values are presented as relative means ± SEM.
