# Supplementary material for: Hepatic Hedgehog signaling contributes to the regulation of IGF1 and IGFBP1 serum levels
Source: Cell Commun Signal. 2014 Feb 18;12:11. doi: 10.1186/1478-811X-12-11 (PMC3946028; doi:10.1186/1478-811X-12-11)
Supplement: Additional file 6: Table S1 — Primers for PCR genotyping of SAC mice. Table S2: Primers for qRT-PCR analyses. Table S3: Primers for RNA interference experiments. Table S4: Primers for Chromatin IP. Table S5: Binding sites of Gli factors and Gli factor-dependent transcription factors to the promoter region of Igf1 and Igfbp-1 in mouse determined by MotifMap. [file 1478-811X-12-11-S6.doc]

**Additional file 6**

**Supporting information Tables**

**Table S1:** Primers for PCR genotyping of SAC mice.

| **Gene** | **Primer** |
| --- | --- |
| *Smo* WT | forward ccactgcgagcctttgcgctac  reverse cccatcacctccgcgtcgca |
| *Smo* flx. | forward atggccgctggccgccccgtg  reverse ggcgctaccggtggatgtgg |
| *Smo* rec. | forward ggcctgcgctgctcaacatgg  reverse ccatcacgtcgaactcctggc |
| Cre-recombinase | forward tcgctgcattaccggtcgatgc  reverse ccatgagtgaacgaacctggtcg |

Genotypes of the transgenic mice were identified by

PCR using primers for the Smo wildtyp (*Smo* WT), the Smo floxed

(*Smo* flx.) allele and the Cre-recombinase.

**Table S2:** Primers for qRT-PCR analyses.

| **Gene** | **Sequence** |
| --- | --- |
| *Acta* | forward ctctcttccagccatctttcat  reverse tataggtggtttcgtggatgc |
| *Arg1* | forward ggcctttgttgatgtcccta  reverse acagaccgtgggttcttcac |
| *beta Actin* | forward catccgtaaagacctctatgccaac  reverse atggagccaccgatccaca |
| *Boc* | forward ccggtacagtgtcaaacagg  reverse cggatggcatgatcaggta |
| *Cdo* | forward gattctgtgctggggtgaa  reverse cagtgggagtccgtgtgat |
| *Cd34* | forward gggtagctctctgcctgatg  reverse tccgtggtagcagaagtcaa |
| *Emr1* | forward ggaggacttctccaagcctatt  reverse aggcctctcagacttctgctt |
| *Fused* | forward tgcctctcagccttcttagg  reverse taagagcgccccatacca |
| *Gck* | forward aacgacccctgcttatcctc  reverse ctgccaggatctgctctacc |
| *Gfap* | forward aagccaagcacgaagctaac  reverse agcaagtgcctcctggtaac |
| *Gli1* | forward cctctcccacatactagaaatctc  reverse accagaaagtccttctgttcc |
| *Gli2* | forward gccaaccagaacaagcag  reverse tctcgtagatgaccacctc |
| *Gli3* | forward ctggcttgattgttcacgag  reverse gcatacatacggtttctcattgg |
| *Gls2* | forward gtatgacttctcgggccagt  reverse tcctgacacagctgacttgg |
| *Hhip1* | forward ctacttgggccagatggaag  reverse ctccaagtaaggctccttgaac |
| *Ihh* | forward gctcacccccaactacaatc  reverse gcggccctcatagtgtaaag |
| *Igf-I* | forward tggatgctcttcagttcgtg  reverse gcaacactcatccacaatgc |
| *Igfbp-1* | forward ctgccaaactgcaacaagaa  reverse tccatgggtagacacaccag |
| *Igfbp-2* | forward cggggccccctggaacatc  reverse ggtattggggttcacacacc |
| *Igfbp-3* | forward aatgtgctgagtcccagagg  reverse ggagcatctactggctctgc |
| *Krt8* | forward agttcgcctccttcattgac  reverse gctgcaacaggctccact |
| *Krt18* | forward gaggcagagattgccaccta  reverse cagtttgcatggagttgctg |
| *Ldha* | forward cctctctgtggcagacttgg  reverse tccgagattccattttgtcc |
| *Krt19* | forward agtcccagctcagcatgaa  reverse taacgggcctccgtctct |
| *Pkm2* | forward gcaggaaccgaagtacgc  reverse tgtgttccaggaaggtgtca |
| *Ptch1* | forward cctcctttacggtggacaaac  reverse atcaactcctcctgccaatg |
| *Ptch2* | forward cttctcccacaagttcatgc  reverse cgatgtcattgttctggtagtcg |
| *Smo* | forward gcaagctcgtgctctggt  reverse gggcatgtagacagcacaca |
| *Shh* | forward tccaaagctcacatccactg  reverse ctccgggacgtaagtccttc |
| *Sufu* | forward cttccagtcagagaacacct  reverse ttgggctgaatgtaactc |

Primers for quantitative real time PCR analyses from hepatocytes, liver tissue and other organs.

**Table S3:** Primers for RNA interference experiments.

| **Gene** | **Sequence** |
| --- | --- |
| Smo | cguagcuuccgggacuaugugcuau  auagcacauagucccggaagcuacg |
| Ptch1 | ccuuccuguucugggagcaauacau  auguauugcucccagaacaggaagg |
| Gli3 | uagcaaggccaucuuggucuucagg  ccugaagaccaagauggccuugcua |

Primers for siRNA knockdown experiments in cultivated hepatocytes.

**Table S4:** Primers for Chromatin IP

| **Gene** | **Sequence** |
| --- | --- |
| *Igf-I* (promotor primer) | forward cagttgccggagggcttaat  reverse cctgccgaggtaacattcca |
| *beta Actin* (exon spanning primer | forward catccgtaaagacctctatgccaac  reverse atggagccaccgatccaca |
| *Rpl30* (intron 2 primer) | SimpleChiP Mouse RPL30 Primer #7015 (Cell signaling) |

Primers for Chromatin Immunoprecipitation in isolated hepatocytes.

**Table S5:** Binding sites of Gli factors and Gli factor-dependent transcription factors to the promoter region of Igf-I and Igfbp-1 in mouse determined by MotifMap

| **Location** | **+/-  strand** | **BBLS** | **BLS** | **NLOD** | **Z-Score** | **FDR** | **Motif ID** | **TF Name** | **Gene** | **Distance  (bp)** | **Region** |
| --- | --- | --- | --- | --- | --- | --- | --- | --- | --- | --- | --- |
| chr10:87320369..87320380 | + | 0,04 | 0,16 | 0,903 | 4,559 | 0,165 | M00220 | SREBP-1 | Igf1 | -3486 | Upstream |
| chr10:87320387..87320394 | + | 1,07 | 2,41 | 1 | 4,156 | 0,108 | M00240 | Nkx2-5 | Igf1 | -3468 | Upstream |
| chr10:87320385..87320396 | + | 0,01 | 0,16 | 0,875 | 4,342 | 0,233 | M00220 | SREBP-1 | Igf1 | -3470 | Upstream |
| chr10:87320479..87320490 | - | 0,03 | 0,74 | 0,916 | 4,462 | 0,153 | M01596 | GLI3 | Igf1 | -3365 | Upstream |
| chr10:87320578..87320584 | + | 2,22 | 2,94 | 1 | 3,683 | 0,179 | M01033 | HNF4 | Igf1 | -3277 | Upstream |
| chr10:87320929..87320945 | + | 0,22 | 1,29 | 0,809 | 4,545 | 0,463 | M00766 | LXR direct repeat 4 | Igf1 | -2926 | Upstream |
| chr10:87321275..87321282 | - | 2,82 | 3,47 | 1 | 3,688 | 0,498 | M01131 | SOX10 | Igf1 | -2573 | Upstream |
| chr10:87321373..87321386 | - | 0,05 | 0,16 | 0,952 | 4,741 | 0,061 | M00764 | HNF4 direct repeat 1 | Igf1 | -2469 | Upstream |
| chr10:87321373..87321387 | + | 0 | 0,16 | 0,9 | 4,309 | 0,241 | M01031 | HNF4 | Igf1 | -2482 | Upstream |
| chr10:87321548..87321554 | - | 3,47 | 4,77 | 1 | 3,683 | 0,191 | M01033 | HNF4 | Igf1 | -2301 | Upstream |
| chr10:87322972..87322978 | - | 2,25 | 3,57 | 1 | 3,683 | 0,18 | M01033 | HNF4 | Igf1 | -877 | Upstream |
| chr10:87323082..87323088 | - | 3,31 | 3,98 | 1 | 3,683 | 0,189 | M01033 | HNF4 | Igf1 | -767 | Upstream |
| chr10:87323449..87323460 | - | 0 | 0,16 | 0,822 | 4,281 | 0,496 | M01702 | GLI1 | Igf1 | -395 | Upstream |
| chr10:87323449..87323460 | - | 0 | 0,16 | 0,854 | 4,416 | 0,457 | M01703 | GLI2 | Igf1 | -395 | Upstream |
| chr10:87323450..87323461 | + | 0,05 | 0,16 | 0,951 | 4,734 | 0,081 | M01596 | GLI3 | Igf1 | -405 | Upstream |
| chr10:87323694..87323700 | + | 2,28 | 3,37 | 1 | 3,591 | 0,046 | M01032 | HNF4 | Igf1 | -161 | Upstream |
| chr10:87323698..87323706 | - | 1,62 | 3,37 | 1 | 4,232 | 0,012 | M00712 | myogenin | Igf1 | -149 | Upstream |
| chr10:87324852..87324858 | + | 2,06 | 3,4 | 1 | 3,683 | 0,177 | M01033 | HNF4 | Igf1 | 997 | Downstream |
| chr10:87325727..87325736 | + | 0,03 | 1,84 | 0,951 | 4,292 | 0,269 | M00223 | STATx | Igf1 | 1872 | Downstream |
| chr10:87325726..87325736 | - | 1,91 | 2,27 | 1 | 4,966 | 0,008 | MA0144 | Stat3 | Igf1 | 1881 | Downstream |
| chr10:87325727..87325737 | + | 1,88 | 2,27 | 0,969 | 4,738 | 0,025 | MA0144 | Stat3 | Igf1 | 1872 | Downstream |
| chr10:87325722..87325738 | - | 0,36 | 1,67 | 0,912 | 4,712 | 0,075 | M01595 | Stat3 | Igf1 | 1883 | Downstream |
| **Location** | **+/-  strand** | **BBLS** | **BLS** | **NLOD** | **Z-Score** | **FDR** | **Motif ID** | **TF Name** | **Gene** | **Distance  (bp)** | **Region** |
| chr10:87325725..87325741 | + | 0,3 | 1,67 | 0,88 | 4,438 | 0,076 | M01595 | Stat3 | Igf1 | 1870 | Downstream |
| chr10:87325721..87325742 | + | 0,09 | 1,36 | 0,796 | 4,549 | 0,259 | M00224 | STAT1 | Igf1 | 1866 | Downstream |
| chr10:87325724..87325746 | + | 0,1 | 1,62 | 0,838 | 4,398 | 0,109 | M01260 | STAT1 | Igf1 | 1869 | Downstream |
| chr10:87326114..87326121 | - | 0,57 | 0,6 | 1 | 3,958 | 0 | MA0087 | SOX5 | Igf1 | 2266 | Downstream |
| chr10:87326128..87326134 | - | 0,72 | 0,79 | 1 | 3,591 | 0,059 | M01032 | HNF4 | Igf1 | 2279 | Downstream |
| chr10:87326237..87326243 | - | 0,64 | 0,79 | 1 | 3,591 | 0,06 | M01032 | HNF4 | Igf1 | 2388 | Downstream |
| chr10:87326238..87326249 | + | 0,02 | 0,16 | 0,905 | 4,72 | 0,414 | M01704 | GLI3 | Igf1 | 2383 | Downstream |
| chr10:87326238..87326249 | + | 0,03 | 0,16 | 0,9 | 4,784 | 0,325 | M01703 | GLI2 | Igf1 | 2383 | Downstream |
| chr10:87326238..87326249 | + | 0,01 | 0,16 | 0,874 | 4,693 | 0,381 | M01702 | GLI1 | Igf1 | 2383 | Downstream |
| chr10:87326373..87326379 | + | 0,74 | 0,79 | 1 | 3,683 | 0,173 | M01033 | HNF4 | Igf1 | 2518 | Downstream |
| chr10:87326624..87326630 | - | 0,61 | 0,79 | 1 | 3,591 | 0,061 | M01032 | HNF4 | Igf1 | 2775 | Downstream |
| chr10:87327411..87327417 | - | 3,15 | 4,42 | 1 | 3,683 | 0,187 | M01033 | HNF4 | Igf1 | 3562 | Downstream |
| chr10:87327820..87327826 | + | 2,92 | 4,17 | 1 | 3,683 | 0,185 | M01033 | HNF4 | Igf1 | 3965 | Downstream |
| chr10:87328479..87328486 | - | 2,49 | 3,34 | 1 | 4,156 | 0,116 | M00240 | Nkx2-5 | Igf1 | 4631 | Downstream |
| chr10:87328589..87328604 | + | 0,78 | 1,6 | 0,926 | 4,535 | 0,119 | M00457 | STAT5A (homodimer) | Igf1 | 4734 | Downstream |
| chr10:87328589..87328604 | + | 0,63 | 1,6 | 0,886 | 4,362 | 0,154 | M00459 | STAT5B (homodimer) | Igf1 | 4734 | Downstream |
| chr10:87328636..87328643 | + | 2,22 | 3,13 | 1 | 4,156 | 0,112 | M00240 | Nkx2-5 | Igf1 | 4781 | Downstream |
|  |  |  |  |  |  |  |  |  |  |  |  |
| chr11:7093324..7093335 | + | 0,02 | 0,48 | 0,892 | 4,277 | 0,28 | M01596 | GLI3 | Igfbp1 | -4465 | Upstream |
| chr11:7093928..7093938 | - | 0,07 | 0,57 | 0,939 | 4,514 | 0,06 | MA0144 | Stat3 | Igfbp1 | -3851 | Upstream |
| chr11:7093929..7093939 | + | 0,02 | 0,57 | 0,912 | 4,319 | 0,12 | MA0144 | Stat3 | Igfbp1 | -3860 | Upstream |
| chr11:7094180..7094186 | + | 0,71 | 0,75 | 1 | 3,683 | 0,17 | M01033 | HNF4 | Igfbp1 | -3609 | Upstream |
| chr11:7094186..7094192 | + | 0,72 | 0,75 | 1 | 3,683 | 0,17 | M01033 | HNF4 | Igfbp1 | -3603 | Upstream |
| chr11:7094210..7094216 | + | 0,75 | 0,75 | 1 | 3,683 | 0,17 | M01033 | HNF4 | Igfbp1 | -3579 | Upstream |
| chr11:7094278..7094284 | - | 0,66 | 0,75 | 1 | 3,683 | 0,17 | M01033 | HNF4 | Igfbp1 | -3505 | Upstream |
| chr11:7094369..7094377 | - | 0,66 | 0,75 | 1 | 3,862 | 0,06 | M00497 | Stat3 | Igfbp1 | -3412 | Upstream |
| chr11:7094443..7094449 | + | 0,71 | 0,75 | 1 | 3,683 | 0,17 | M01033 | HNF4 | Igfbp1 | -3346 | Upstream |
| **Location** | **+/-  strand** | **BBLS** | **BLS** | **NLOD** | **Z-Score** | **FDR** | **Motif ID** | **TF Name** | **Gene** | **Distance  (bp)** | **Region** |
| chr11:7094900..7094915 | - | 0,14 | 0,55 | 0,984 | 4,533 | 0,23 | M00131 | HNF3beta | Igfbp1 | -2874 | Upstream |
| chr11:7094912..7094919 | + | 1,26 | 1,53 | 1 | 4,156 | 0,11 | M00240 | Nkx2-5 | Igfbp1 | -2877 | Upstream |
| chr11:7094945..7094952 | + | 0,87 | 1,53 | 1 | 4,156 | 0,11 | M00240 | Nkx2-5 | Igfbp1 | -2844 | Upstream |
| chr11:7095446..7095452 | - | 0,58 | 0,58 | 1 | 3,591 | 0,06 | M01032 | HNF4 | Igfbp1 | -2337 | Upstream |
| chr11:7096716..7096722 | - | 0,15 | 0,16 | 1 | 3,591 | 0,06 | M01032 | HNF4 | Igfbp1 | -1067 | Upstream |
| chr11:7096722..7096728 | - | 0,15 | 0,16 | 1 | 3,591 | 0,06 | M01032 | HNF4 | Igfbp1 | -1061 | Upstream |
| chr11:7096958..7096964 | + | 0,59 | 0,67 | 1 | 3,591 | 0,06 | M01032 | HNF4 | Igfbp1 | -831 | Upstream |
| chr11:7097725..7097740 | - | 1,85 | 2,77 | 0,942 | 4,88 | 0,21 | M00132 | HNF1 | Igfbp1 | -49 | Upstream |
| chr11:7097723..7097741 | - | 1,46 | 2,77 | 0,899 | 4,696 | 0,27 | M00790 | HNF1 | Igfbp1 | -48 | Upstream |
| chr11:7097722..7097743 | + | 0,97 | 2,77 | 0,881 | 4,617 | 0,21 | M01011 | HNF1 | Igfbp1 | -67 | Upstream |
| chr11:7098757..7098763 | - | 2,4 | 3,29 | 1 | 3,683 | 0,18 | M01033 | HNF4 | Igfbp1 | 974 | Downstream |
| chr11:7099109..7099116 | + | 1,35 | 2,89 | 1 | 4,156 | 0,11 | M00240 | Nkx2-5 | Igfbp1 | 1320 | Downstream |
| chr11:7099199..7099210 | + | 0 | 0,48 | 0,846 | 4,347 | 0,48 | M01703 | GLI2 | Igfbp1 | 1410 | Downstream |
| chr11:7099271..7099279 | - | 1,17 | 1,51 | 1 | 3,726 | 0,15 | M00500 | STAT6 | Igfbp1 | 1490 | Downstream |
| chr11:7100313..7100319 | + | 1,36 | 1,76 | 1 | 3,683 | 0,17 | M01033 | HNF4 | Igfbp1 | 2524 | Downstream |
| chr11:7101411..7101417 | + | 0,73 | 1,1 | 1 | 3,591 | 0,06 | M01032 | HNF4 | Igfbp1 | 3622 | Downstream |
| chr11:7101709..7101715 | + | 1,18 | 1,49 | 1 | 3,591 | 0,05 | M01032 | HNF4 | Igfbp1 | 3920 | Downstream |
| chr11:7101831..7101842 | + | 0,02 | 0,57 | 0,909 | 4,406 | 0,2 | M01596 | GLI3 | Igfbp1 | 4042 | Downstream |
| chr11:7101865..7101871 | - | 4,9 | 7,54 | 1 | 3,591 | 0,06 | M01032 | HNF4 | Igfbp1 | 4082 | Downstream |

The promoter region was scanned from -5000 bp to 5000 bp relative to the transcription start site. Bayesian Branch Length Score (BBLS), Branch Length Score (BLS), Normalized Log-Odds score (NLOD), False Discovery Rate (FDR).
