# Supplementary material for: Hepatic Hedgehog signaling contributes to the regulation of IGF1 and IGFBP1 serum levels
Source: Cell Commun Signal. 2014 Feb 18;12:11. doi: 10.1186/1478-811X-12-11 (PMC3946028; doi:10.1186/1478-811X-12-11)
Supplement: Additional file 7: Figure S6 — Correlation analyses of IGF-I serum concentrations and body weight from male and female transgenic SAC mice. Correlation analyses of IGF-I serum concentrations and body weight from (A): male SAC-WT (black circles) (n = 7) mice and SAC-KO (open circles) (n = 6) mice and (B): female SAC-WT (black circles) (n = 9) mice and SAC-KO (open circles) (n = 8) mice. c = correlation coefficient; p = significance level. [file 1478-811X-12-11-S7.pdf]

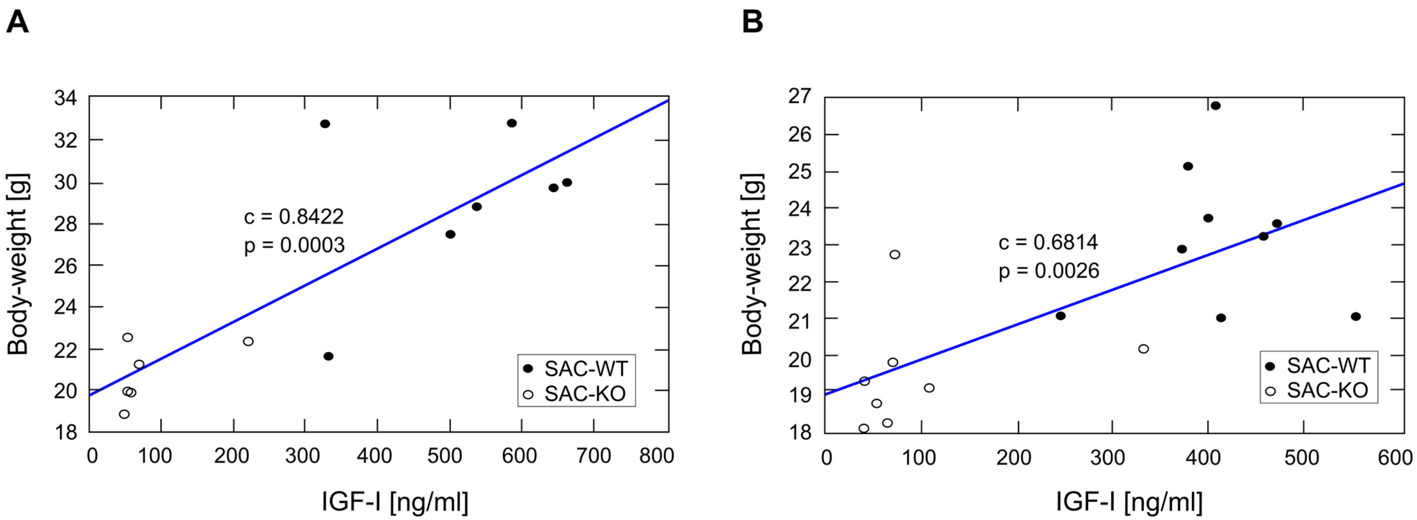

Figure S6: Correlation analyses of IGF-I serum concentrations and body weight from male and female transgenic SAC mice.

Correlation analyses of IGF-I serum concentrations and body weight from **(A):** male SAC-WT (black circles) (n=7) mice and SAC-KO (open circles) (n=6) mice and **(B):** female SAC-WT (black circles) (n=9) mice and SAC-KO (open circles) (n=8) mice. c=correlation coefficient; p=significance level.
